# Measurement of Glycosylated Alpha-Fetoprotein Improves Diagnostic Power over the Native Form in Hepatocellular Carcinoma

**DOI:** 10.1371/journal.pone.0110366

**Published:** 2014-10-13

**Authors:** Hyunsoo Kim, Kyunggon Kim, Jonghwa Jin, Jiyoung Park, Su Jong Yu, Jung-Hwan Yoon, Youngsoo Kim

**Affiliations:** 1 Department of Biomedical Sciences, Seoul National University College of Medicine, Seoul, Republic of Korea; 2 Department of Biomedical Engineering, Seoul National University College of Medicine, Seoul, Republic of Korea; 3 Department of Internal Medicine, Seoul National University College of Medicine, Seoul, Republic of Korea; Xiangya Hospital of Central South University, China

## Abstract

Serum alpha-fetoprotein (AFP) has long been used as a diagnostic marker for hepatocellular carcinoma (HCC), albeit controversially. Although it remains widely used in clinics, the value of AFP in HCC diagnosis has recently been challenged due to its significant rates of false positive and false negative findings. To improve the efficacy of AFP as HCC diagnostic marker, we developed a method of measuring total and glycosylated AFP by multiple reaction monitoring (MRM)-MS. In this study, we verified the total amount of AFP (nonglycopeptide levels) and the degree of glycosylated AFP (deglycopeptide levels) in 60 normal (41 men and 19 women; mean age 53 years; range 32–74 years), 35 LC (23 men and 12 women; mean age 56 years; range 43–78 years; HBV-related), and 60 HCC subjects (42 men and 18 women; mean age 58 years; range 38–76 years; HBV-related; 30 stage I, 15 stage II, and 10 stage III). By MRM-MS analysis, the nonglycopeptide had 56.7% sensitivity, 68.3% specificity, and an AUC of 0.687 [cutoff value: ≥0.02 (light/heavy ratio)], comparing the normal and HCC group, whereas the deglycopeptide had 93.3% sensitivity, 68.3% specificity, and an AUC of 0.859 [cutoff value: ≥0.02 (light/heavy ratio)]. In comparing the stage I HCC subgroup with the LC group, the nonglycopeptide had a sensitivity of 66.7%, specificity of 80.0%, and an AUC of 0.712 [cutoff value: ≥0.02 (light/heavy ratio)], whereas the deglycopeptide had a sensitivity of 96.7%, specificity of 80.0%, and an AUC of 0.918 [cutoff value: ≥0.02 (light/heavy ratio)]. These data demonstrate that the discriminatory power of the deglycopeptide is greater than that of the nonglycopeptide. We conclude that deglycopeptide can distinguish cancer status between normal subjects and HCC patients better than nonglycopeptide.

## Introduction

Glycosylation is one of the most important and common post-translational modifications (PTMs) of proteins that are secreted into serum. Glycosylation influences many functional aspects of proteins, including their structure and functions [1]. The expression and degree of glycosylation is significantly altered by various diseases, such as cancer, and thus, glycoproteins are associated with abnormal phenomena in patients with cancer [2]–[4]. To this end, quantitative measurements of glycoproteins might be useful in discovering biomarkers for cancer.

The carbohydrates on glycosylated protein biomarkers undergo modifications in cancer. For example, the carbohydrate moieties of AFP are altered in cancer, and such changes are considered to be more useful markers of HCC [5]. Studies from the past several decades have demonstrated that total AFP is a collection of heterogeneous glycoproteins that can be fractionated by affinity electrophoresis into 3 glycoforms–AFP-L1, AFP-L2, and AFP-L3–based on their reactivity with the lectin Lens culinaris agglutinin (LCA). AFP-L3 binds strongly to LCA through an α1-6 bond between its additional fucose and the reducing terminus of N-acetylglucosamine, in contrast to AFP-L1 [6]–[8].

AFP levels are sometimes elevated in patients with chronic hepatitis and cirrhosis who have no evidence of HCC [9]. AFP has a reported sensitivity of 39% to 65% and a specificity of 65% to 94%; approximately one-third of early-stage HCC patients with small tumors (<3 cm) have normal AFP levels [10], [11]. Thus, clinicians are dissatisfied with AFP as a marker due to its high false-positive and false-negative rates [12].

Multiple reaction monitoring (MRM)-based quantification using triple quadrupole mass spectrometry (MS) is especially useful for measuring glycosylated biomarkers. MRM-MS, a multiplexed, targeted proteomic platform, is a rapid and cost-effective approach for measuring protein biomarkers for preclinical verification [13]. Nonglycopeptides (unmodified native peptides) and glycopeptides (glycosylated peptides) of glycoprotein markers are valuable biomarkers for the diagnosis and prediction of diseases, because their expression levels and degree of glycosylation reflect quantitative differences of disease states, as in cancer.

In this study, we measured total AFP and glycosylated AFP by MRM-MS. Total AFP was represented by common nonglycopeptides among all forms of AFP, and glycosylated AFP comprised the portion of deglycosylated peptides after treatment of glycosylated peptides with PNGase F. Measurement of the deglycopeptide fraction from the glycosylated AFP yielded better AUC values than the nonglycopeptides of total AFP. Consequently, on measuring AFP concentrations in serum from HCC patients versus normal healthy controls and early-stage HCC versus liver cirrhosis by MRM-MS, AFP deglycopeptides had greater power in distinguishing, compared with nonglycopeptides.

Notably, total AFP and glycosylated AFP were measured effectively by MRM-MS in the form of nonglycopeptides and deglycopeptides, respectively, improving our diagnosis of HCC versus normal and early-stage HCC versus LC serum. In addition, MRM-MS is a platform that improves the measurement of total AFP and glycosylated AFP in glycoprotein biomarker assays, which is more advantageous compared with conventional methods, such as antibody-based measurements by lectin affinity electrophoresis and liquid-phase binding assays.

## Materials and Methods

### Materials

Standard glycoprotein (origin: yeast) was purchased from Sigma-Aldrich (St. Louis, MO). Trypsin was obtained from Promega (Madison, WI). Peptide-N-glycosidase F (PNGase F) was purchased from New England Biolabs (NEB, Beverly, MA). HPLC-grade water and acetonitrile were obtained from Thermo Fisher Scientific (Bremen, Germany). Serum depletion was performed for the 6 most abundant proteins using a multiple affinity removal system (MARS), consisting of an LC column (Agilent, 5185–5984); buffer A for sample loading, washes, and equilibration (Agilent, 5185–5987); and buffer B for elution (Agilent, 5185–5988). Stable isotope-labeled peptides [isotopically labeled (^13^C and ^15^N) amino acids] were obtained from JPT (Berlin, Germany).

### Clinical sample information

The institutional review board (IRB) of Seoul National University Hospital (approval No. H-1103-056-355) approved the study protocol, and written informed consent was obtained from each patient or legally authorized representative. The clinical characteristics of the patients are shown in Table 1.

**Table 1:** Characteristics of clinical subjects for MRM-MS analysis.

	Normal group	LC group	HCC group
**Total patient number**	60	35	60
**Gender (Male/Female)**	41/19	23/12	42/18
**Age (Mean, Range)**	53 (32–74)	56 (43–78)	58 (38–76)
**Etiology of liver disease**		HBV, 35 (100%)	HBV, 60 (100%)
**AFP value (mean, range)**			
<20 ng/ml	60	35	26
20–400 ng/ml	0	0	18
>400 ng/ml	0	0	16
**Albumin (g/dL)**	4.3±0.18	4.2±0.3	3.6±0.5
**Bilirubin (mg/dL)**	1.3±0.4	1.4±0.7	1.3±0.8
**AST (IU/L)**	22.5±6.0	32.3±24.3	89.1±142.3
**ALT (IU/L)**	20.2±8.3	37.6±35.0	97.1±249.9
**ALP (IU/L)**	41.7±19.2	78.4±20.6	123.5±77.6
**HBV DNA levels (IU/mL)** a			
Not detected		11	1
Detected (<10^3^/units)		9	3
Detected (≥10^3^/units)		6	25
**Antiviral therapy** b			
Yes (%)		23 (65.7%)	17 (28.3%)
No (%)		12 (34.3%)	43 (71.7%)
**Treatment type**			
Surgical resection			1
RFA			3
PEIT			22
TACE			30
TACE & PEIT			4
**Tumor size (cm)** c			
<2			21
2∼5			16
>5			3
**Tumor stage** d			
I			30
II			15
III			10
IV			0
**Target lesion response**			
CR			38
PR			19
SD			3
PD			0

**Abbreviations**

TACE: Transcatheter arterial chemoembolization.

PEIT: Percutaneous ethanol injection therapy.

RFA: Radiofrequency ablation.

AFP: Alpha-fetoprotein.

ALT: Alanine aminotransferase; AST: Aspartate aminotransferase; ALP: Alkaline phosphatase.

CR: Complete Response; PR: Partial Response; SD: Stable disease; PD: Progressive disease.

Albumin, Bilirubin, AST, ALT, ALP, and HBV DNA levels data are presented as mean ± SD.

a HBV DNA levels were provided for 26 among a total of 35 liver cirrhosis patients, and 29 among the 60 HCC patients.

b Antiviral therapy was treatment with Entecavir, Tenofovir, Zeffix, Hepsera and Revovir.

c Tumor size was provided for 40 among a total of 60 HCC patients.

d According to American Joint Committee on Cancer (AJCC) staging system (7^th^ edition, 2010).

The clinical sample set comprised healthy controls (n = 60), patients with liver cirrhosis (n = 35), and patients with hepatocellular carcinoma (n = 60). The healthy control group (normal group) comprised sixty healthy volunteers who visited the Healthcare Center of Seoul National University Hospital. All control subjects were confirmed, based on normal liver function test results, including serum alanine and aspartate aminotransferases, and were negative for hepatitis B virus surface antigen and anti-hepatitis C virus. Liver ultrasonography was performed to screen for fatty liver disease, and all healthy controls had normal findings.

The liver cirrhosis group (LC group) included 35 patients with compensated HBV cirrhosis and no HCC. The cirrhosis group had at least 1 year of follow-up from the time that serum was obtained for these studies. Patients were diagnosed with cirrhosis, based on established clinical, laboratory, and imaging criteria with ultrasound examination.

Sixty patients before HCC treatment who were infected with hepatitis B virus (HBV) were also enrolled, from whom serum samples were collected and defined as the HCC group. The diagnosis of HCC was made per the American Association for the Study of Liver Diseases by a hepatologist with more than 20 years of experience [14]. To reduce causal heterogeneity, HCC patients who had other types of chronic liver disease, except chronic hepatitis B, such as chronic hepatitis C and alcoholic hepatitis, were excluded.

HCC stage was classified per the tumor-node-metastasis (TNM) staging system (7^th^ edition, 2010, the American Joint Committee on Cancer staging system). TNM staging of the 55 cases demonstrated stage I in 30 cases, stage II in 15 cases, stage III in 10 cases, and stage IV in 0 cases. Insufficient information was available to assign stage in 5 HCC cases.

All subjects (n = 155) were recruited during the study period from September 2005 to August 2012 and collected by the Liver Research Institute, Seoul National University College of Medicine. Blood samples were centrifuged immediately at 3000 rpm for 10 min at 4°C to fractionate the serum. The resulting supernatant was aliquoted (100 µL) and stored at −80°C until analysis.

### Sample size calculations

We calculate the sample size that was needed for clinical MRM verification with reference to previous studies using AUC (area under the curve) [15]. The sample size was calculated, based on an AUC with a type I error rate α of 0.05 and type II error rate β of 0.10 (90% power). To anticipate similar AUC values as in previous studies, we needed a sample size of 29 each in the normal and HCC groups, each totaling 58, and 24 each in the HCC and recovery groups, totaling 48. A minimum sample size of 29 per group was necessary for the MRM assays to determine a significant difference between groups (Table S1).

### Standard glycoprotein preparation

Standard glycoprotein (INV1) was prepared to 100 µg/µL. Standard glycoprotein samples were denatured with 6 M urea, 100 mM Tris, pH 8.0, and 20 mM dithiothreitol (DTT) at 37°C for 60 min and alkylated with 50 mM iodoacetamide (IAA) at room temperature in the dark for 30 min. The urea was diluted 15-fold with 100 mM Tris, pH 8.0. One INV1 sample was deglycosylated with 2 µL of PNGase F (500,000 units/mL) at 37°C for 12 h and incubated and digested in a solution of 1∶50 trypsin (w/w) at 37°C for 16 h. The other INV1 sample was incubated in 2 µL 100 mM Tris, pH 8.0 (untreated PNGase F) at 37°C for 12 h and digested as described above. The 2 INV1 digests were dried on a speed vacuum, diluted in mobile phase A, and spiked with stable isotope-labeled standard (SIS) peptide, as needed.

### Clinical serum sample preparation

The 6 most abundant proteins in human serum (albumin, transferrin, IgG, IgA, haptoglobin, and α1-antitrypsin) were depleted on an HPLC system (Shimadzu Co., Kyoto, Japan) that was equipped with a multiple affinity removal system LC column (Agilent Technologies, Santa Clara, CA). Crude human serum samples were diluted by a factor of 5 with buffer A and passed through 0.22-µm filters by centrifugation (12,000 g, room temperature, 2–3 min). Diluted crude serum was injected at 0.25 mL/min, and flowthrough fractions were collected and stored at −80°C. Depleted serum samples were concentrated by centrifugal filtration using a 3000-Da molecular weight cutoff (MWCO) (Millipore, Bedford, MA). Concentrated serum protein was quantified by bicinchoninic acid (BCA) assay.

Aliquots of serum samples (100 µg) were denatured and digested as described above. Tryptic digestion was stopped with formic acid (FA) at a final concentration of 1% and desalted on OASIS HLB 1-cc (30 mg) extraction cartridges (Waters Corp., Milford, MA). The cartridges were equilibrated sequentially with 3 mL acetonitrile (ACN) and 5 mL water/0.1% FA prior to loading of the tryptic digestions. The cartridges were washed with 3 mL water/0.1% FA and eluted with 1 mL of 60% ACN/0.1% FA. The eluted samples were frozen and lyophilized on a speed vacuum. Before MRM-MS analysis, the samples were reconstituted in mobile phase A to 1 µg/µL.

### MRM-MS transition (Q1/Q3) selection

Skyline software was used to generate a list of all possible b-, y- series fragment ions for 2^+^ precursor ion charge states, spanning the m/z range from 300 to 1400. In brief, full-length protein sequences were imported into Skyline in FASTA format and designed into peptides, each with a list of product ions for monitoring by MRM-MS. In selecting transitions through Skyline, the peptide filter condition was as follows: maximum length of peptide of 24, including at least 6 amino acids.

Peptides with repeat arginines (Arg, R) or lysines (Lys, K) were discarded. If methionine (Met, M) was included in the peptide, it was discarded to avoid the risk of modification. If proline (Pro, P) was next to arginine (Arg, R) or lysine (Lys, K), the peptide was discarded. If a peptide contained histidine (His, H), it was discarded to avoid the risk of charge alterations. Peptides that satisfied these conditions were used as Q1 transitions. Next, we selected a maximum of 10 Q3 transitions from the fragmentation ions that were derived from the Q1 transitions in descending order.

For glycopeptides, theoretical transition values were selected, based on the original FASTA sequences after changing asparagine (Asn, N) in the NxS/T motif into aspartic acid (Asp, D), using Skyline. In addition, to verify that the measured peptides originated from the endogenous peptide that was tested, a stable isotope-labeled standard (SIS) peptide was used. The sequence of the SIS peptide is identical to that of the measured peptide but has ^13^C and ^15^N in the C-terminal arginine (Arg, R) and lysine (Lys, K). This result is described in Table S2.

### Quantification by multiple reaction monitoring

An Agilent 1260 Infinity HPLC system was used to inject 5 µL of digestion samples directly into a reversed phase analytical column (150 mm×0.5 mm i.d., Agilent Zorbax SB-C18, 3.5-µm particle size) that was maintained at 40°C. Mobile phase A consisted of water/0.1% FA, and mobile phase B comprised ACN/0.1% FA. The peptides were separated and eluted at 20 µL/min on a linear gradient of mobile phase B from 3% to 40% B in 45 min. The gradient was ramped to 70% B for 5 min and 3% B for 10 min to equilibrate the column for the next run. The total LC run time was 60 min.

The MRM-MS data were analyzed using ESI on an Agilent 6490 triple quadrupole (QQQ) mass spectrometer (Agilent Technologies, Santa Clara, CA) that was equipped with an iFunnel Technology source and controlled by MassHunter Workstation software (Agilent, B.06.00). The MRM-MS analysis was conducted in the positive ion mode with the ion spray capillary voltage and nozzle voltage set to 2500 and 2000 V, respectively. The drying gas temperature was set to 250°C at 15 L/min, and the sheath gas temperature was 350°C at 12 L/min. The nebulizer was set to 30 psi, the fragmentor voltage was 380 V, and the cell accelerator voltage was 5 V. For the MRM-MS acquisition, delta EMV was set to 200 V. Quadrupoles 1 and 3 were maintained at unit (0.7 FWHM) resolution.

### Collision energy optimization

The initial collision energy (CE) linear equation was derived from our optimized experiments, prior to the Skyline CE optimization step, using 600 stable isotope-labeled standard (SIS) peptides that were measured (data not shown). In the CE optimization module in Skyline, “Step count” was set to 5 on either side of the equation-predicted value, and “Step size” was set to 2V. The b and y ions for 2^+^ precursor ion charge states were used, and in total, 11 collision energy voltage values were considered for each fragment ion. The maximum number of concurrent measurements was set to 132. The data were acquired and imported into Skyline for peak area integration, which was reviewed manually and finalized by a single investigator.

### Study design for blocking and randomization

Blocking and randomization can prevent the negative impacts of nonbiologic effects on molecular biomarker discovery [16]. In our experiments, nonbiologic effects could have been introduced during the sample preparation (order of MARS depletion and order of tryptic digestion) and MRM-MS analysis (order of injection). Three step where the experimenter’s subjectivity could have led to bias of sample groups was negated by blocked randomization method.

We applied the blocked randomization design when assigning the sample group to remove confounding nonbiologic effects. There were equal numbers of cases (HCC group) and controls (normal group) in every block, with random block size. We then assigned each group of order to a random permutation of the samples in the corresponding group (Table S3). Blocked randomization was performed using Excel (2013, Microsoft) and Random Allocation (version 1.00, University of Medical Sciences).

### Statistical analysis of MRM-MS data

To analyze the MRM-MS data, raw MRM-MS data files were processed in Skyline. To increase the accuracy of the peak area integration, we manually confirmed and corrected the wrong automatic assignments for each targeted peak area. In our peak integration step, we used the Savitzky-Golay smoothing algorithm. Differences were analyzed by *T*-test between the normal versus HCC group and the LC versus stage I HCC subgroup. To evaluate the discriminatory power of the serum biomarkers between groups, we analyzed the receiver operator characteristic (ROC) curves and generated interactive plots. ROC curves were compared using DeLong’s method [17]. All statistical analyses were performed using MedCalc (Mariakerke, Belgium, version 12.2.1).

## Results

### Overall scheme

We performed a combined measurement in a single run of the total AFP concentration and glycosylated AFP fraction after PNGase F treatment. There are 3 glycosylated forms of AFP–AFP-L1, L2, and L3–based on its reactivity to Lens culinaris agglutinin (LCA) by affinity electrophoresis (Figure S1A). The concentration of total AFP and the N-linked glycosylated AFP fraction (AFP-L1, L2 and L3) that can be cleaved by PNGase F (Figure S1B) could be measured by MRM-MS in the same MRM run.

**Figure 1:**
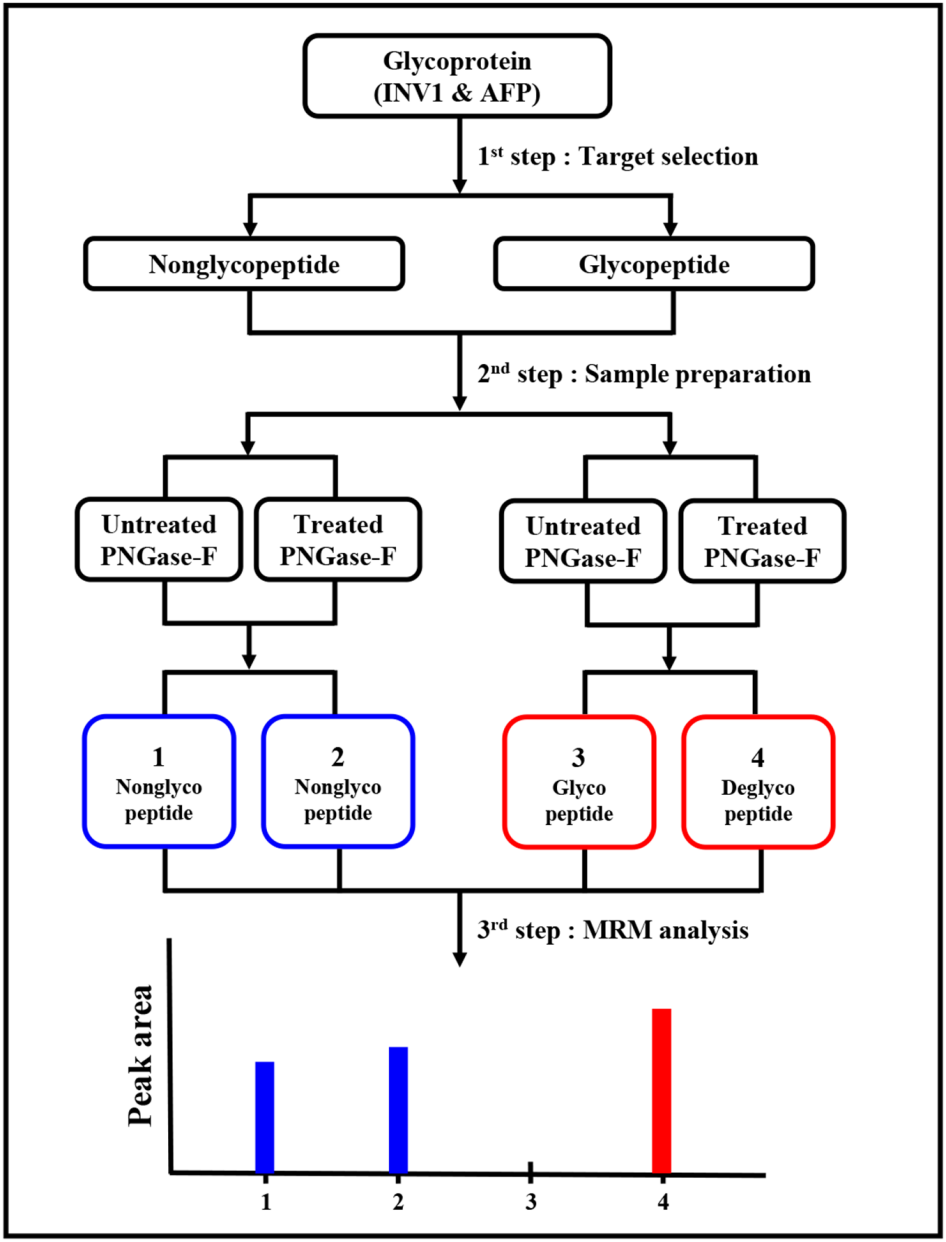
Development of MRM-MS method for measuring glycoproteins. To develop the MRM-MS method for measuring nonglycopeptides, glycopeptides, and deglycopeptides, we determined whether our MRM-MS approach was suitable for measuring glycoproteins using a standard glycoprotein, such as yeast invertase 1 (INV1), and applied the MRM-MS method to measure the alpha-fetoprotein (AFP) in human serum samples.

To develop an MRM-MS method for measuring glycoprotein concentration, such as AFP, we measured a standard glycoprotein of INV1 to determine whether MRM-MS was suitable for measuring glycoprotein concentrations and applied MRM-MS to measure AFP concentrations (Figure 1). The total AFP concentration was measured, based on nonglycopeptides, whereas the glycosyated AFP fraction was represented by the corresponding deglycopeptide that was generated by PNGase F treatment. Consequently, the discriminatory power was examined for total AFP and glycosylated AFP in serum samples from normal healthy controls versus HCC patients and liver cirrhosis versus early-stage HCC patients.

### Establishment of MRM-MS measurement using standard glycoprotein

We first determine whether MRM-MS is suitable for measuring glycoproteins using standard glycoproteins, such as yeast INV1. Four peptides were examined in developing and assessing the MRM-MS method (Figure S2A and Table S2): 2 peptides (IEIYSSDDLK and VVDFGK) were nonglycopeptides, and 2 were glycopeptides (NPVLAA*N*STQFR and FAT*N*TTLTK).

In examining their ability to be detected, all nonglycopeptides and deglycopeptides (SIS standard: Asn to Asp) coeluted with the corresponding SIS heavy peptides, whereas glycopeptides did not coelute with its SIS heavy peptide form (SIS standard: Asn with no glycan). Because the SIS heavy peptides were synthesized only using Asn with no glycan, the SIS heavy peptide that represents the glycopeptide comprises only amino acid residues; thus, the existence of a glycopeptide is measured alternatively in the form as in the MRM-MS (eg, glycosylated Asn is measured as Asn or Asp). We measured the 4 peptides using the corresponding SIS heavy peptides, as detailed in Figure S3A–H. Because we could not use glycosylated SIS heavy peptides for the 2 glycopeptides, we measured the SIS heavy peptides NPVLAA*D*STQFR and FAT*D*TTLTK indirectly as substitutes for the glycopeptides NPVLAA*N*STQFR and FAT*N*TTLTK (Figure S3E & S3G).

Two nonglycopeptides (IEIYSSDDLK; Figure S3A & S3B, and VVDFGK; Figure S3C & S3D) were detected in the PNGase F-untreated (S3A, S3C) and PNGase F-treated (S3B, S3D) samples; the 2 nonglycopeptides had slightly greater intensity with PNGase F treatment (Figure S4). It is possible that PNGase F removes the glycan of a peptide, which may effect the absence of steric hindrance due to the glycan, allowing trypsin approach to a target peptide more easily. Consequently this accessibility might increase digestion and affect the peak intensity of MS/MS spectra [18].

The 2 glycopeptides (NPVLAA*N*STQFR and FAT*N*TTLTK) were detected only with PNGage F treatment as peaks of the corresponding deglycopeptides (NPVLAA*D*STQFR; Figure S3F, and FAT*D*TTLTK; Figure S3H). In the PNGase F-untreated, no extracted ion chromatogram (XIC) was detected for the endogenous glycopeptide forms, whereas SIS heavy peptides were detected with unglycosylated Asn (NPVLAA*N*STQFR; Figure S3E, and FAT*N*TTLTK; Figure S3G). In the MRM-MS analysis, glycopeptides could not be detected, because they were measured, based on the original sequence–ie, NPVLAA*N*STQFR and FAT*N*TTLTK, respectively. Conversely, the corresponding deglycosylated sequences for PNGage F-treated glycopeptides–NPVLAA*D*STQFR and FAT*D*TTLTK–were detected as MRM peaks for the glycosylated peptides of NPVLAA*N*STQFR and FAT*N*TTLTK, respectively.

To develop the quantitative method, we evaluated the linearity of nonglycopeptides, glycopeptides, and deglycopeptides that originated from INV1 by analyzing the standard curves of serial dilutions for known concentrations of SIS heavy peptides. To generate calibration curves for the 3 types of peptides, SIS heavy peptides were serially diluted (0, 4, 13, 40, 120, and 370 fmol), with the light peptides as internal standards. Digested light yeast peptide (370 nmol) was added to each diluted sample. Each experiment was repeated in triplicate to generate coefficients of variation (%CV) and calibration curve values (*R* ^2^).

The calibration curves demonstrated linearity over more than 2 orders of magnitude for the concentration ranges and strong linear correlation (*R* ^2^>0.99) in all 3 peptide forms (Figure S5A–D). By MRM-MS analysis using the standard peptides, we developed the MRM-MS method for measuring glycoprotein concentrations. Notably, the PNGase F-untreated endogenous light glycopeptides (NPVLAA*N*STQFR and FAT*N*TTLTK, filled downward arrows) did not generate peaks from MRM, as shown in Figure S5C–D.

### Preliminary MRM-MS using pooled serum samples

Prior to MRM-MS of AFP using individual samples, we first selected predictable transitions using Skyline, in which the N-linked glycopeptide included the NxS/T motif, whereas the nonglycopeptide did not. Table 2 shows 3 AFP peptides that were used in the MRM-MS; the sequences of the nonglycopeptide, glycopeptide, and deglycopeptide of AFP were GYQELLEK, V*N*FTEIQK, and V*D*FTEIQK, respectively. For MRM-MS of the 3 AFP peptides, we determined their detectability in the preliminary MRM-MS run.

**Table 2:** AFP peptides and their parameters for MRM-MS.

Peptide type	Peptide sequence	Q1 (m/z)	Q1 ion charge	Q3 (m/z)	Q3 ion charge	Q3 ion type	Retention time (min)	Isotope	Fragmentor (volt)	Initial CE (volt)	Optimized CE (volt)
Nonglycopeptide	GYQELLEK	490.3	2	759.4	1	y6	23.6	light	380	17.3	11.3
				631.4	1	y5	23.6	light	380	17.3	13.3
				502.3	1	y4	23.6	light	380	17.3	15.3
				389.2	1	y3	23.6	light	380	17.3	17.3
				276.2	1	y2	23.6	light	380	17.3	21.3
				221.1	1	b2	23.6	light	380	17.3	11.3
				349.2	1	b3	23.6	light	380	17.3	9.3
				591.3	1	b5	23.6	light	380	17.3	11.3
				704.4	1	b6	23.6	light	380	17.3	7.3
				833.4	1	b7	23.6	light	380	17.3	9.3
	GYQELLEK	494.3	2	767.4	1	y6	23.6	heavy	380	17.3	11.3
				639.4	1	y5	23.6	heavy	380	17.3	13.3
				510.3	1	y4	23.6	heavy	380	17.3	15.3
				397.3	1	y3	23.6	heavy	380	17.3	17.3
				284.2	1	y2	23.6	heavy	380	17.3	21.3
				221.1	1	b2	23.6	heavy	380	17.3	11.3
				349.2	1	b3	23.6	heavy	380	17.3	9.3
				591.3	1	b5	23.6	heavy	380	17.3	11.3
				704.4	1	b6	23.6	heavy	380	17.3	7.3
				833.4	1	b7	23.6	heavy	380	17.3	9.3
Glycopeptide^a^	V*N*FTEIQK	489.8	2	879.5	1	y7	22.0	light	380	17.3	13.3
				765.4	1	y6	22.0	light	380	17.3	13.3
				618.3	1	y5	22.0	light	380	17.3	15.3
				517.3	1	y4	22.0	light	380	17.3	11.3
				388.3	1	y3	22.0	light	380	17.3	21.3
				275.2	1	y2	22.0	light	380	17.3	21.3
				361.2	1	b3	22.0	light	380	17.3	9.3
				462.2	1	b4	22.0	light	380	17.3	9.3
				591.3	1	b5	22.0	light	380	17.3	11.3
				704.4	1	b6	22.0	light	380	17.3	9.3
				832.4	1	b7	22.0	light	380	17.3	7.3
	V*N*FTEIQK	493.8	2	887.5	1	y7	22.0	heavy	380	17.3	13.3
				773.4	1	y6	22.0	heavy	380	17.3	13.3
				626.4	1	y5	22.0	heavy	380	17.3	15.3
				525.3	1	y4	22.0	heavy	380	17.3	11.3
				396.3	1	y3	22.0	heavy	380	17.3	21.3
				283.2	1	y2	22.0	heavy	380	17.3	21.3
				361.2	1	b3	22.0	heavy	380	17.3	9.3
				462.2	1	b4	22.0	heavy	380	17.3	9.3
				591.3	1	b5	22.0	heavy	380	17.3	11.3
				704.4	1	b6	22.0	heavy	380	17.3	9.3
				832.4	1	b7	22.0	heavy	380	17.3	7.3
Deglycopeptide	V*D*FTEIQK	490.3	2	880.4	1	y7	22.7	light	380	17.3	13.3
				765.4	1	y6	22.7	light	380	17.3	13.3
				618.3	1	y5	22.7	light	380	17.3	15.3
				517.3	1	y4	22.7	light	380	17.3	11.3
				388.3	1	y3	22.7	light	380	17.3	21.3
				275.2	1	y2	22.7	light	380	17.3	21.3
				362.2	1	b3	22.7	light	380	17.3	9.3
				463.2	1	b4	22.7	light	380	17.3	9.3
				592.3	1	b5	22.7	light	380	17.3	11.3
				705.3	1	b6	22.7	light	380	17.3	9.3
				833.4	1	b7	22.7	light	380	17.3	7.3
	V*D*FTEIQK	494.3	2	888.5	1	y7	22.7	heavy	380	17.3	13.3
				773.4	1	y6	22.7	heavy	380	17.3	13.3
				626.4	1	y5	22.7	heavy	380	17.3	15.3
				525.3	1	y4	22.7	heavy	380	17.3	11.3
				396.3	1	y3	22.7	heavy	380	17.3	21.3
				283.2	1	y2	22.7	heavy	380	17.3	21.3
				362.2	1	b3	22.7	heavy	380	17.3	9.3
				463.2	1	b4	22.7	heavy	380	17.3	9.3
				592.3	1	b5	22.7	heavy	380	17.3	11.3
				705.3	1	b6	22.7	heavy	380	17.3	9.3
				833.4	1	b7	22.7	heavy	380	17.3	7.3

a The molecular mass of glycopeptide is estimated based on Asn with no glycan moiety.

Nonglycopeptides (GYQELLEK) and deglycopeptide (V*D*FTEIQK) of AFP coeluted with the endogenous light and SIS heavy peptides at the same retention time. In brief, the nonglycopeptide (GYQELLEK) was coeluted in the PNGase F-untreated (Figure S6A) and PNGase F-treated (Figure S6B) conditions. However, the glycopeptide was not detected (Figure S6C) when the PNGase F-untreated glycosylated peptide was analyzed by the glycopeptide form with the no-glycan form (V*N*FTEIQK). The PNGage F-treated glycosylated peptide coeluted (Figure S6D) with the deglycopeptide form (V*D*FTEIQK).

**Figure 2:**
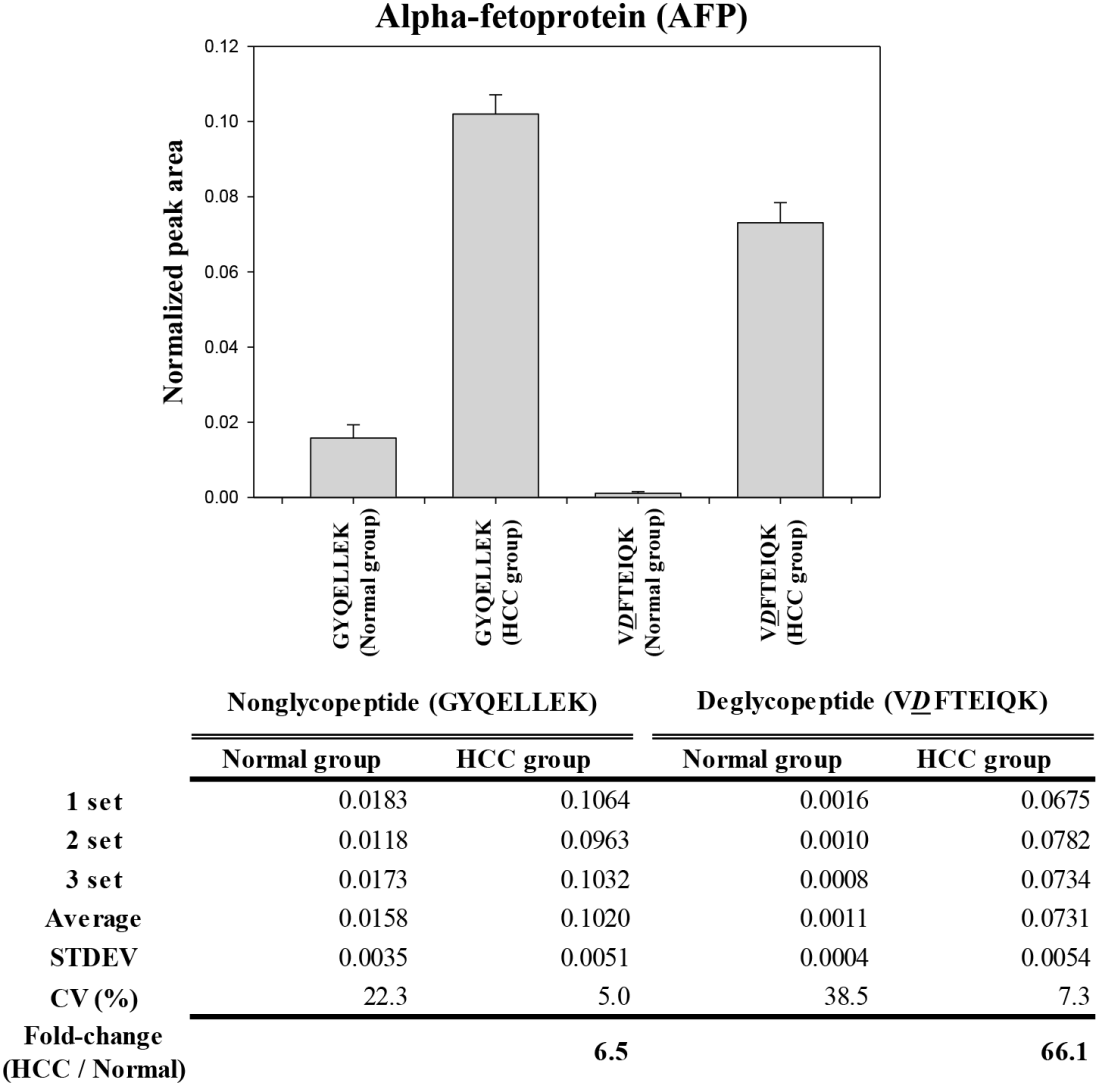
Preliminary MRM-MS analysis using pooled serum samples. Normal samples (n = 20) and HCC samples (n = 20) were pooled separately and analyzed by MRM-MS using the nonglycopeptide (GYQELLEK) and deglycopeptide (V*D*FTEIQK).

Serum AFP concentrations rise in HCC patients versus normal subjects [19]–[22]; thus, we examined this difference between the normal healthy and HCC group using MRM-MS measurements. 20 normal samples and 20 HCC samples were pooled separately and analyzed by MRM-MS using the nonglycopeptide (GYQELLEK). The MS/MS intensity for the nonglycopeptide increased in the normal versus HCC group (Figure 2).

With regard to measuring glycopeptide concentrations, because the glycosylated glycopeptide form (V*N*FTEIQK) could not be detected directly using MRM-MS, we analyzed the deglycopeptide form (V*D*FTEIQK), which was generated by treating V*N*FTEIQK with PNGase F. The normalized peak area for the nonglycopeptide and deglycopeptide increased in HCC patients versus the normal group (Figure 2). Particularly, the peak area ratio of HCC to the normal group was 6.5 and 66.5 for the nonglycopeptide and deglycopeptide, respectively, indicating that total AFP concentration is a useful index for liver cancer diagnostics and that measuring the fraction of glycosylated AFP that has been treated by PNGase F is a more important element in HCC diagnosis.

### Linearity of calibration curve for SIS AFP peptide

**Figure 3:**
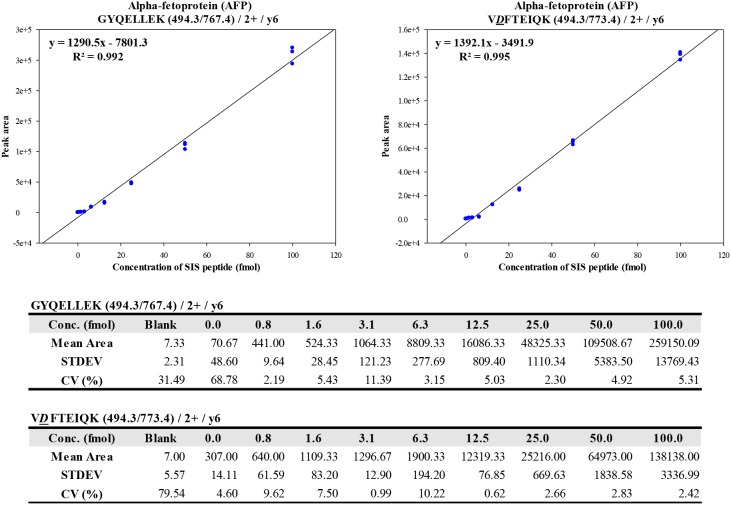
Generation of calibration curve for nonglycopeptide (GYQELLEK) and deglycopeptide (V*D*FTEIQK) of AFP. To generate a calibration curve for the nonglycopeptide and deglycopeptide, SIS heavy peptides were serially diluted (9 concentration points: 0.0, 0.8, 1.6, 3.1, 6.3, 12.5, 25.0, 50.0, and 100.0 fmol) with the endogenous light peptide as an internal standard (pooled serum: 5 µg), added to each serially diluted sample. Each experiment was performed in triplicate to generate coefficients of variation (%CV) and calibration curve values (*R* ^2^).

In the quantitative MRM-MS analysis, we determined the linearity of the nonglycopeptide (GYQELLEK) and deglycopeptide (V*D*FTEIQK) with regard to AFP, which was performed by generating a standard curve for the nonglycopeptide and deglycopeptide, composed of serial dilutions for known concentrations of SIS heavy peptides. SIS heavy peptides were serially diluted (9 concentrations: 0.0, 0.8, 1.6, 3.1, 6.3, 12.5, 25.0, 50.0, and 100.0 fmol) with the endogenous light peptide as an internal standard (pooled serum: 5 µg), added to each serially diluted sample. Each experiment was repeated in triplicate to generate coefficients of variation (%CV) and calibration curve values (*R* ^2^). The calibration curves were linear over more than 2 orders of magnitude in concentration, wherein the nonglycopeptide (GYQELLEK) and deglycopeptide (V*D*FTEIQ) showed strong linearity (R^2^>0.99) (Figure 3).

### MRM-MS measurements of AFP peptides using individual serum samples

To measure the nonglycopeptide (GYQELLEK) and deglycopeptide (V*D*FTEIQK) of AFP using MRM-MS in individual normal and HCC serum samples, we determined the optimal spiking concentrations for SIS heavy peptides that minimized the measurement errors for the peak area ratio between endogenous light peptide and SIS heavy peptide. By MRM analysis using pooled samples, which comprised endogenous light (pooled serum: 5 µg) and serially diluted SIS heavy peptides (0–200 fmol), we determined the optimal range of SIS heavy peptide for spiking (peak area ratio of light peptide to heavy peptide = 1). The concentrations of SIS heavy peptides for the nonglycopeptide (GYQELLEK) and deglycopeptide (V*D*FTEIQK) that were used to spike were 10.3 and 7.3 fmol, respectively.

Individual serum samples were analyzed using the MRM-MS measurements with the spiked SIS heavy peptide mixture of nonglycopeptide (GYQELLEK) and deglycopeptide (V*D*FTEIQK) as the internal standard. Based on the MRM measurements using 155 individual serum samples, we measured the concentrations of the nonglycopeptide (GYQELLEK) and deglycopeptide (V*D*FTEIQK) of AFP in the normal (n = 60), LC group (n = 35) and HCC group (n = 60). The MRM measurements were imported into Skyline, and the peak areas of each transition were calculated, after normalization by the peak area of the spiked SIS heavy peptide (Figure S7). Next, the relative quantities of each transition were compared between the nonglycopeptide (GYQELLEK) and deglycopeptide (V*D*FTEIQK) in the normal versus HCC group and the LC versus stage I HCC subgroup.

**Figure 4:**
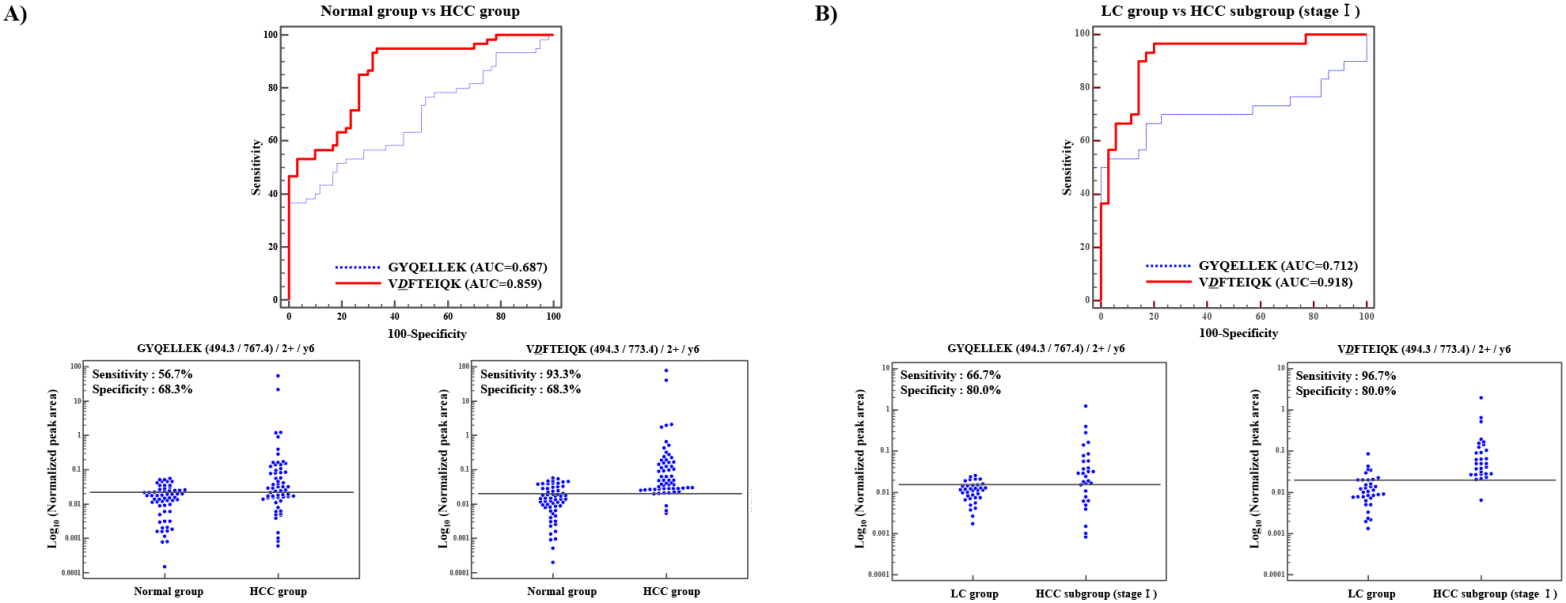
Receiver operating characteristic (ROC) curves and interactive plots for the nonglycopeptide (GYQELLEK) and deglycopeptide (V*D*FTEIQK) of AFP, respectively. The normalized peak areas of transitions were compared between normal and HCC group. In the interactive plots, sensitivity was calculated based on a specificity of 68.3%, which was calculated per an AFP cutoff value of 20 ng/mL (56.7% sensitivity), representing significant prognostic impact for HCC (A) LC was compared to Stage I HCC subgroup, in the interactive plots, sensitivity was calculated based on a specificity of 80.0% which was calculated with optimal deglycopeptide level (B).

To determine the efficacy of serum biomarkers in distinguishing HCC versus normal controls and the stage I HCC subgroup versus LC group, we drew receiver operator characteristic (ROC) curves and interactive plots, and the MRM-MS data on the nonglycopeptide (GYQELLEK) and deglycopeptide (V*D*FTEIQK) from both groups were analyzed. (Figure 4).

HCC group was compared to normal control group, the nonglycopeptide had a sensitivity of 56.7%, specificity of 68.3%, AUC of 0.687 [cutoff value: ≥0.02 (light/heavy ratio)] whereas the deglycopeptide had sensitivity of 93.3%, specificity of 68.3%, AUC of 0.859 [cutoff value: ≥0.02 (light/heavy ratio)] (Figure 4A).

In comparing the stage I HCC subgroup with the LC group, the nonglycopeptide had a sensitivity of 66.7%, specificity of 80.0%, and AUC of 0.712 [cutoff value: ≥0.02 (light/heavy ratio)], whereas the deglycopeptide had a sensitivity of 96.7%, specificity of 80.0%, and AUC of 0.918 [cutoff value: ≥0.02 (light/heavy ratio)] (Figure 4B). Thus, the discriminatory power of the deglycopeptide was greater versus the nonglycopeptide.

Pairwise differences in AUC values between nonglycopeptide and deglycopeptide estimations were analyzed by DeLong test [17]–the difference in AUC values was significant (normal vs HCC: *P*-value = 0.0010; LC vs stage I HCC: *P*-value = 0.0042) (Table S4).

Notably, considering that the nonglycopeptide (GYQELLEK) is measuring total AFP, it would be more advantageous to measure the deglycopeptide (V*D*FTEIQK) or take a combined measurement of nonglycopeptide (GYQELLEK) and deglycopeptide (V*D*FTEIQK), which could improve the diagnostic power in HCC.

## Discussion

Most protein biomarkers are based on the premise that native proteins are differentially expressed in normal versus disease states. Recent studies have also demonstrated that protein biomarkers can discriminate such states according to the degree of glycosylation of native proteins [23]–[28].

In this study, we performed MRM measurements for 2 types of peptides (nonglycopeptide and deglycopeptide) that target a glycoprotein and serum AFP, wherein the amount of nonglycopeptide represents the total glycoprotein concentration and the deglycopeptide represents the glycosylated fraction of the glycoprotein. Based on our hypothesis, we compared the total AFP concentration (represented as nonglycopeptide: GYQELLEK) with the glycosylated AFP fraction (represented as deglycopeptide: V*D*FTEIQK) between the normal, LC, and HCC groups.

To quantitatively analyze the total AFP and glycosylated AFP, respectively, we established an MRM-MS method for measuring the nonglycopeptide and deglycopeptide that corresponded to the glycosylated glycopeptide in serum samples. In developing this method, we first assessed whether the MRM-MS approach was suitable for measuring glycoprotein levels. We observed that MRM-MS measured the nonglycopeptide and deglycopeptide as alternatives to total AFP concentration and the glycosylated AFP fraction that was cleaved by PNGase F, respectively. Then, we performed MRM measurements for the endogenous light peptides and the SIS heavy peptides that coeluted with them as internal standards.

The nonglycopeptide was detected in the PNGase F-untreated and PNGase F-treated conditions, whereas the glycosylated glycopeptide was seen only after PNGase F treatment in the deglycopeptide form, because the original amino acid sequence with glycan could not be detected by MRM-MS. Specifically, although the PNGase F-treated deglycosylated state could be measured based on the deglycopeptide sequence (Asn changed to Asp), no glycopeptide could be detected by MRM in the glycosylated state (PNGase F-untreated). Thus, this sequence-based approach to the MRM measurements is useful in measuring the glycoprotein concentrations.

AFP is a significant marker for the clinical diagnosis and evaluation of suspected HCC patients. As a diagnostic tool for HCC, AFP level is determined by immunoenzymatic chemiluminescence; the cutoff of serum AFP levels for significant prognostic impact for HCC is 20 ng/mL (AFP-negative: <20 ng/mL and AFP-positive: ≥20 ng/mL) [22].

However, some reports have demonstrated that AFP level (cutoff value: ≥20 ng/mL) is a poor diagnostic tool in HCC, with a sensitivity of 54%. For example, 46%, 36%, and 18% of 1158 HCC patients had normal (<20 ng/mL), elevated (20–400 ng/mL), and diagnostic AFP levels (>400 ng/mL), respectively [29], [30]. We noted the similar trend in AFP level in our HCC patients–as shown in Table 1, 43%, 30%, and 27% of 60 HCC patients had normal (<20 ng/mL), elevated (20–400 ng/mL), and diagnostic levels of AFP (>400 ng/mL), respectively (yielding a sensitivity of 56.7%). This result suggests that solely using total AFP level is not an effective method for distinguishing HCC from healthy subjects.

There are other biomarkers besides AFP that can be used to screen for HCC, such as DCP, also known as prothrombin induced by vitamin K absence II (PIVKA II). DCP is an abnormal product of liver carboxylation during thrombogen formation. The serum level of DCP in patients with HCC is significantly higher than in healthy adults and patients with nonmalignant hepatopathy (chronic hepatitis and cirrhosis) [31], [32].

DCP can be used as a prognostic indicator for patients with small HCC tumors. High serum levels of DCP are also associated with a greater risk of HCC recurrence and worse overall survival in patients with an HCC tumor under 3 cm [33], [34]. DCP can be used to evaluate the prognosis of patients with small HCC tumors but remains insufficient in the primary screening of HCC patients [35].

Recently, AFP-L3 (%) has been used as an additional indicator of total AFP in the diagnosis of HCC, demonstrating superior performance compared with measuring total AFP alone [36]–[41]. Similarly, we developed a MRM-based measurement approach using a deglycopeptide of AFP as an alternative to the glycosylated AFP fraction (AFP-L1, L2 and L3). By MRM-MS, the nonglycopeptide had a sensitivity of 56.7%, specificity of 68.3%, and AUC of 0.687 in distinguishing normal and HCC subjects versus 93.3%, 68.3%, and 0.859, respectively with the deglycopeptide. Also, the nonglycopeptide had a sensitivity of 66.7%, specificity of 80.0%, and AUC of 0.712 in distinguishing LC and the stage I HCC subgroup versus 96.7%, 80.0%, and 0.918, respectively, with the deglycopeptide. Thus, the discriminatory power of the deglycopeptide was better than that of the nonglycopeptide (Figure 4). To compare the nonglycopeptide and deglycopeptide of AFP accurately, we fixed their specificity in calculating the sensitivity.

In total, 30 HCC patients were primarily TNM stage I–ie, the early stage of HCC–suggesting that these findings can be applied to clinical settings in discriminating early-stage HCC from LC. Thus, deglycopeptide levels can differentiate small tumors from cirrhotic liver, which is significant, because other markers can not distinguish between early-stage HCC and LC. Our data indicate that upregulated deglycopeptide levels in early-stage HCC patients function in tumorigenesis and can be used as a marker for the early detection of a cirrhotic liver that progresses to HCC.

Our MRM-MS-based method has benefits in verifying glycoprotein biomarkers in human samples, because it does not require any complex or irreproducible glycoprotein enrichment steps. Further, determining the amount and extent of glycosylation in glycoproteins is difficult through conventional methods. Specifically, the differences in expression and degree of glycosylation of AFP have not been compared using antibody-based assays, such as western blot and ELISA (enzyme-linked immunosorbent assay).

Ultimately, 2 types of peptide markers–a nonglycopeptide and deglycopeptide–were used to distinguish HCC from normal controls and early-stage HCC from the LC group. Further verification of their value in larger samples should facilitate the development of better biomarkers for HCC.

## Supporting Information

Figure S1 (PDF)

Figure S2 (PDF)

Figure S3 (PDF)

Figure S4 (PDF)

Figure S5 (PDF)

Figure S6 (PDF)

Figure S7 (PDF)

Table S1 (XLSX)

Table S2 (XLSX)

Table S3 (XLSX)

Table S4 (XLSX)
