# Supplementary material for: Measurement of Glycosylated Alpha-Fetoprotein Improves Diagnostic Power over the Native Form in Hepatocellular Carcinoma
Source: PLoS One. 2014 Oct 13;9(10):e110366. doi: 10.1371/journal.pone.0110366 (PMC4195728; doi:10.1371/journal.pone.0110366)
Supplement: Figure S1 — (PDF) [file pone.0110366.s001.pdf]

Figure S1. Three glycosylated AFP forms (L1, L2, and L3) and cleavage pattern of peptide-N-glycosidase F (PNGase F).

Total AFP can be separated into 3 subspecies—AFP-L1, L2, and L3—based on its reactivity to Lens culinaris agglutinin (LCA) on affinity electrophoresis. AFP-L1 does not react with LCA. AFP-L3 is the LCA-bound fraction of AFP **(A)**. PNGase F is an amidase that cleaves between the GlcNAc and asparagine residues (Asn, N) of high-mannose, hybrid, and complex oligosaccharides from N-linked glycoproteins. Conversely, PNGase F is unable to cleave N-linked glycans from glycoproteins when the GlcNAc residue is linked to an α1-3 fucose residue **(B)**.

A)

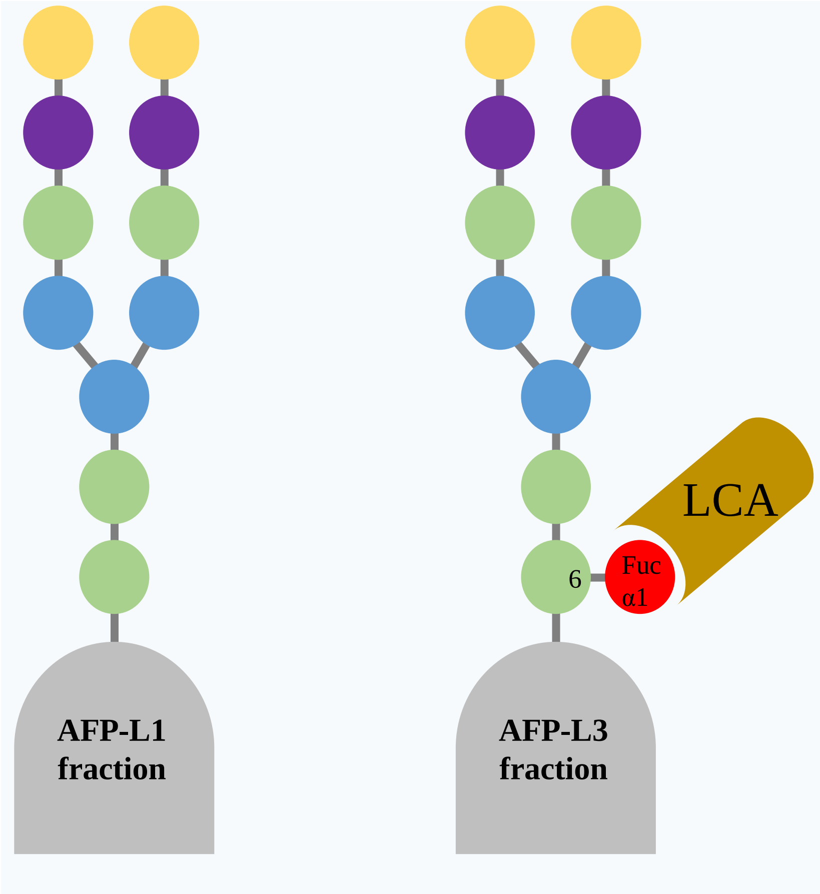

B)

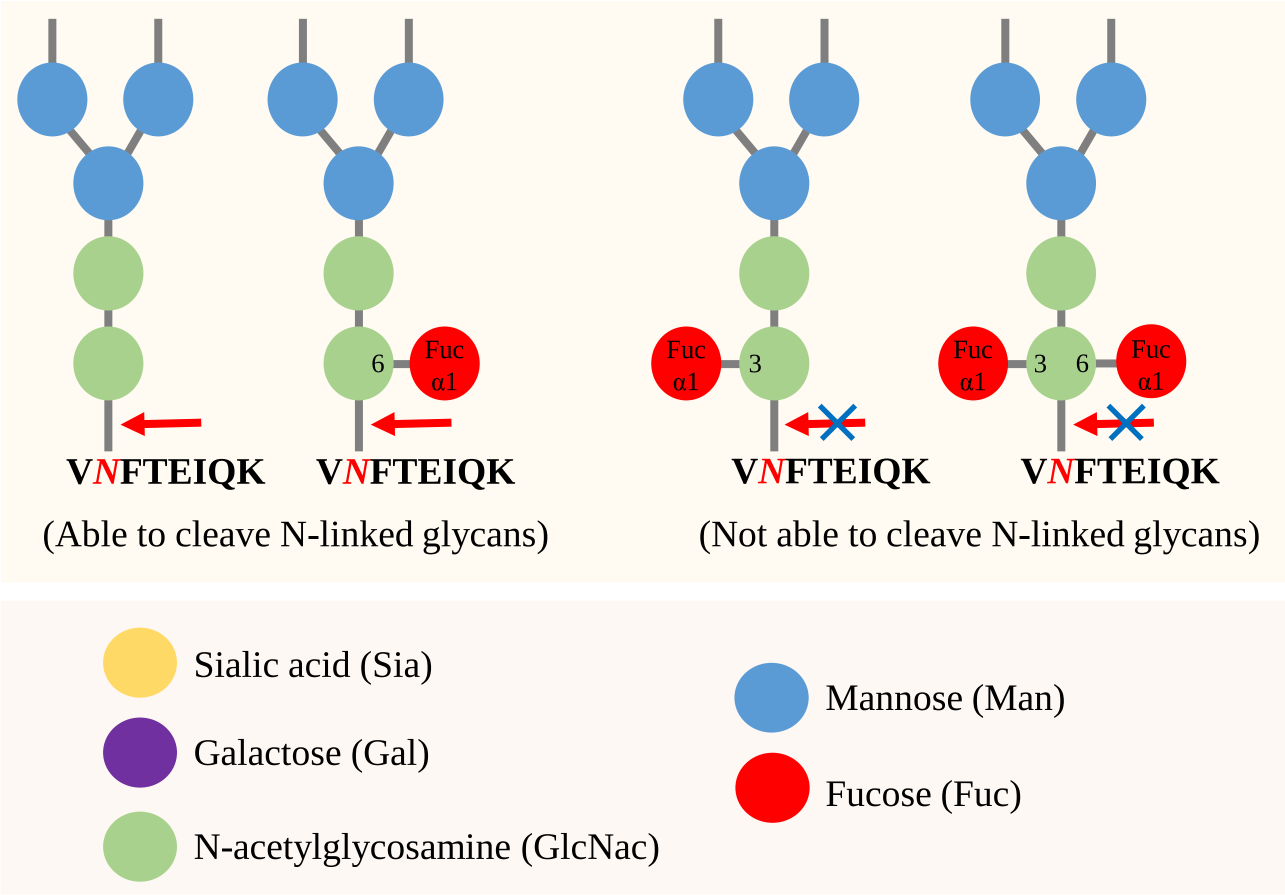
6
Fuc
α1
VNFTEIQK
VNFTEIQK
(Able to cleave N-linked glycans)
Fuc
α1
3
Fuc
α1
3
6
Fuc
α1
VNFTEIQK
VNFTEIQK
(Not able to cleave N-linked glycans)
Sialic acid (Sia)
Galactose (Gal)
N-acetylglycosamine (GlcNac)
Mannose (Man)
Fucose (Fuc)
