# Supplementary material for: Measurement of Glycosylated Alpha-Fetoprotein Improves Diagnostic Power over the Native Form in Hepatocellular Carcinoma
Source: PLoS One. 2014 Oct 13;9(10):e110366. doi: 10.1371/journal.pone.0110366 (PMC4195728; doi:10.1371/journal.pone.0110366)
Supplement: Figure S2 — (PDF) [file pone.0110366.s002.pdf]

Figure S2. Full sequence of standard glycoprotein invertase 1 (INV1, yeast) (A) and alpha-fetoprotein (AFP, human) (B).

Glycopeptide is in italics and underlined; N-glycosylation sites are labeled red. Nonglycopeptide is in italics and bold.

A) Invertase 1 (INV1) / Saccharomyces cerevisiae (yeast)

MLLQAFLFLLAGFAAKISASMTNETSDRPLVHFTPNKGWMNDPNGLWYDAKEGKWHLYFQYNPNDTVWGL
PLFWGHATSDDLTHWQDEPVAIAPKRKDSGAYSGSMVIDYNNTSGFFNDTIDPRQRCVAIWTYNTPESEEQYIS
YSLDGGYTFTEYQK*NPVLAANSTQFR*DPKVFWYEPSKKWIMTAAKSQDYK***IEIYSSDDLK***SWKLESAFANEGF
LGYQYECPGLIEVPSEQDPSKSHWVMFISINPGAPAGGSFNQYFVGSFNGHHFEAFDNQSR***VVDFGK***DYYALQ
TFFNTDPTYGSALGIAWASNWEYSAFVPSNPWRSSMSLVRPFSLNTEYQANPETELINLKAEPILNISSAGPWSR
*FATNTTLTK*ANSYNVDLSNSTGTLEFELVYAVNTTQTISKSVFADLSLWFKGLEDPEEYLRMGFEVSASSFFLDR
GNSKVKFVKENPYFTNRMSVNNQPFKSENDLSYYKVYGLLDQNILELYFNDGDVVSTNTYFMTTGNALGSV
NMTTGVDNLFYIDKFQVREVK

B) Alpha-fetoprotein (AFP) / Homo sapiens (human)

MKWVESIFLIFLLNFTESRTLHRNEYGIASILDSYQCTAEISLADLATIFFAQFVQEATYKEVSKMVKDALTAIEK
PTGDEQSSGCLENQLPAFLEELCHEKEILEKYGHSDCCSQSEEGRHNCFLAHKKPTPASIPLFQVPEPVTSCEAY
EEDRETFMNKFIYEIARRHPFLYAPTILLWAARYDKIIPSCCKAENAVECFQTKAATVTKELRESSLLNQHACAV
MKNFGTRTFQAITVTKLSQKFTK*VNFTEIQ*KLVLDVAHVHEHCCRGDVLDCLQDGEKIMSYICSQQDTLSNKIT
ECCKLTTLERGQCIIHAENDEKPEGLSPNLNRFLGDRDFNQFSSGEKNIFLASFVHEYSRRHPQLAVSVILRVAK
GYQELLEKCFQTENPLECQDKGEEELQKYIQESQALAKRSCGLFQKLGEYYLQNAFLVAYTKKAPQLTSSELM
AITRKMAATAATCCQLSEDKLLACGEGAADIIIGHLCIRHEMTPVNPGVGQCCTSSYANRRPCFSSLVVDETYV
PPAFSDDKFIFHKDLCQAQGVALQTMKQEFLINLVKQKPQITEEQLEAVIADFSGLLEKCCQGQEQEVCFAEEG
QKLISKTRAALGV
