# Supplementary material for: Measurement of Glycosylated Alpha-Fetoprotein Improves Diagnostic Power over the Native Form in Hepatocellular Carcinoma
Source: PLoS One. 2014 Oct 13;9(10):e110366. doi: 10.1371/journal.pone.0110366 (PMC4195728; doi:10.1371/journal.pone.0110366)
Supplement: Figure S3 — (PDF) [file pone.0110366.s003.pdf]

Figure S3. Confirmation of detectability for nonglycopeptides, glycopeptides, and deglycopeptides.

Two nonglycopeptides were treated with PNGase F or not, wherein the endogenous light peptides and corresponding SIS heavy peptides coeluted at the same retention time and the transitions were well overlaid **(A-D)**. For the glycopeptide, the endogenous light peptides did not coelute with their corresponding SIS heavy peptides **(E and G)**. For the deglycopeptide, the endogenous light peptides coeluted with their corresponding SIS heavy peptides **(F and H)**. In the MRM-MS analysis, all nonglycopeptides and deglycopeptides were detected as endogenous light and SIS heavy peptides that coeluted, whereas glycopeptides were detected only in their SIS heavy peptide form.

A)

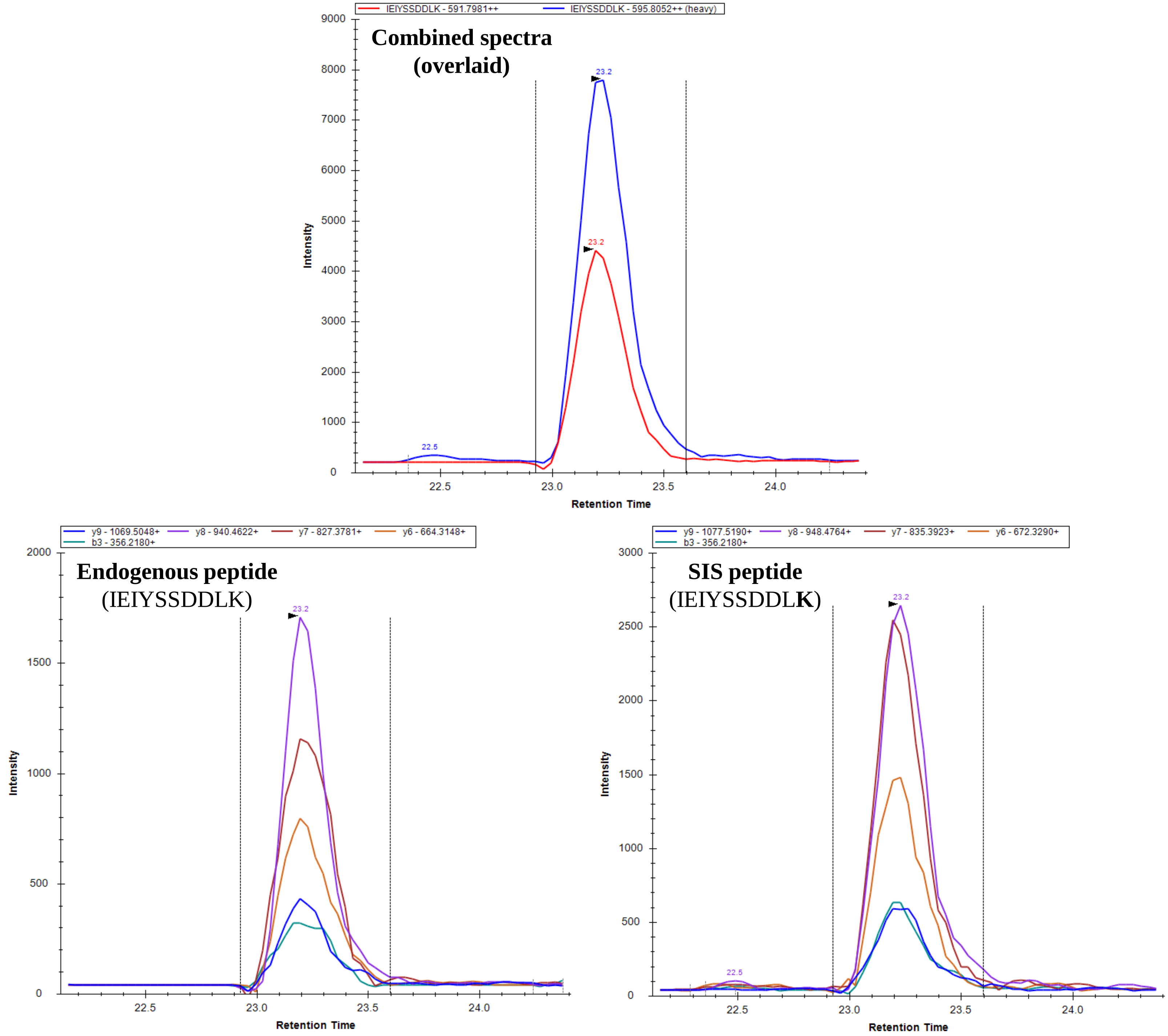

IEIYSSDDLK - 591.7981++
IEIYSSDDLK - 595.8052++ (heavy)
Combined spectra
(overlaid)
Intensity
Retention Time
Endogenous peptide
(IEIYSSDDLK)
y9 - 1069.5048+
y8 - 940.4622+
y7 - 827.3781+
y6 - 664.3148+
b3 - 356.2180+
SIS peptide
(IEIYSSDDLK)
y9 - 1077.5190+
y8 - 948.4764+
y7 - 835.3923+
y6 - 672.3290+
b3 - 356.2180+

B)

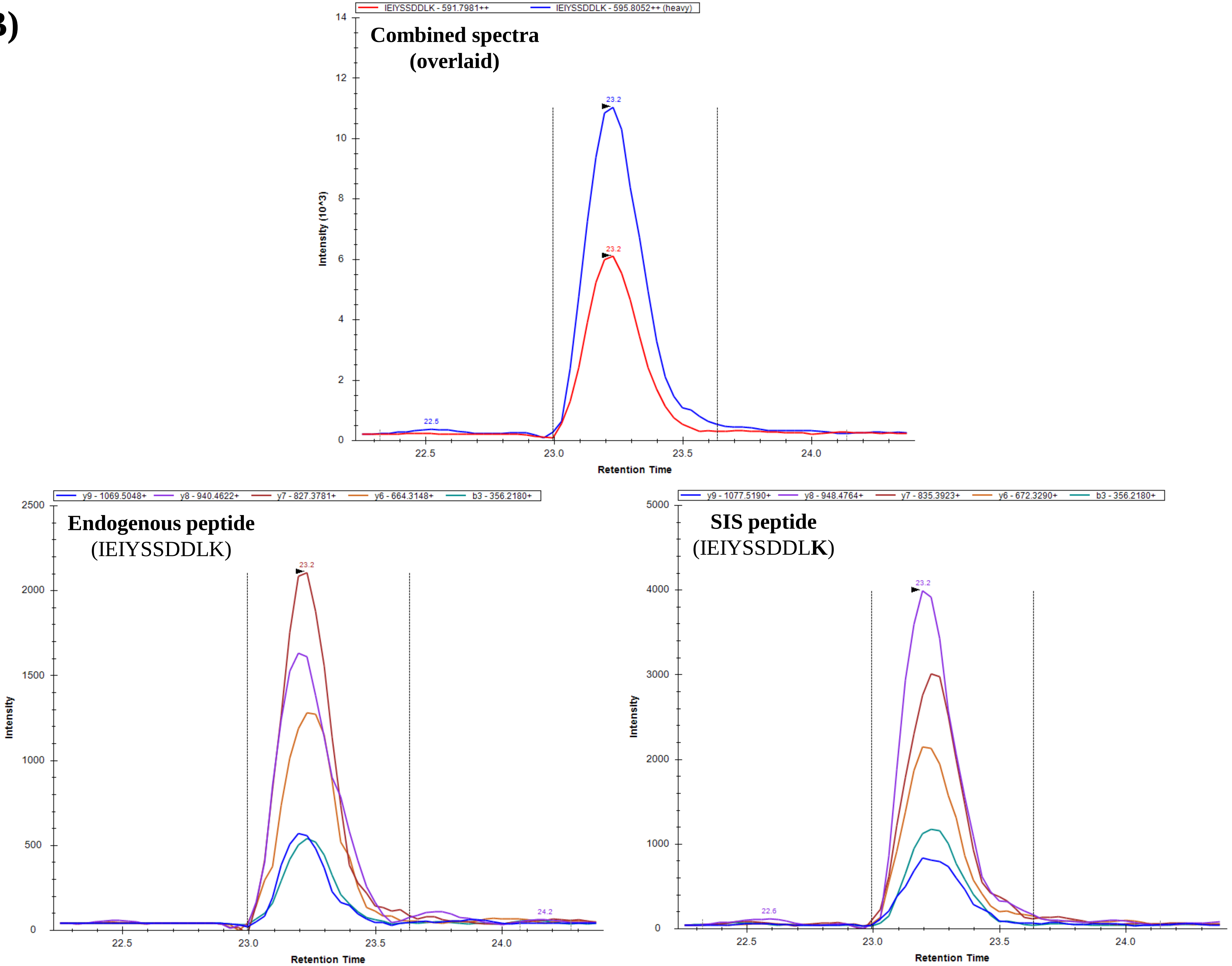
IEIYSSDDLK - 591.7981++
IEIYSSDDLK - 595.8052++ (heavy)
Combined spectra (overlaid)
Intensity (10^3)
Retention Time
y9 - 1069.5048+
y8 - 940.4622+
y7 - 827.3781+
y6 - 664.3148+
b3 - 356.2180+
Endogenous peptide (IEIYSSDDLK)
Intensity
Retention Time
y9 - 1077.5190+
y8 - 948.4764+
y7 - 835.3923+
y6 - 672.3290+
b3 - 356.2180+
SIS peptide (IEIYSSDDL**K**)
Intensity
Retention Time

C)

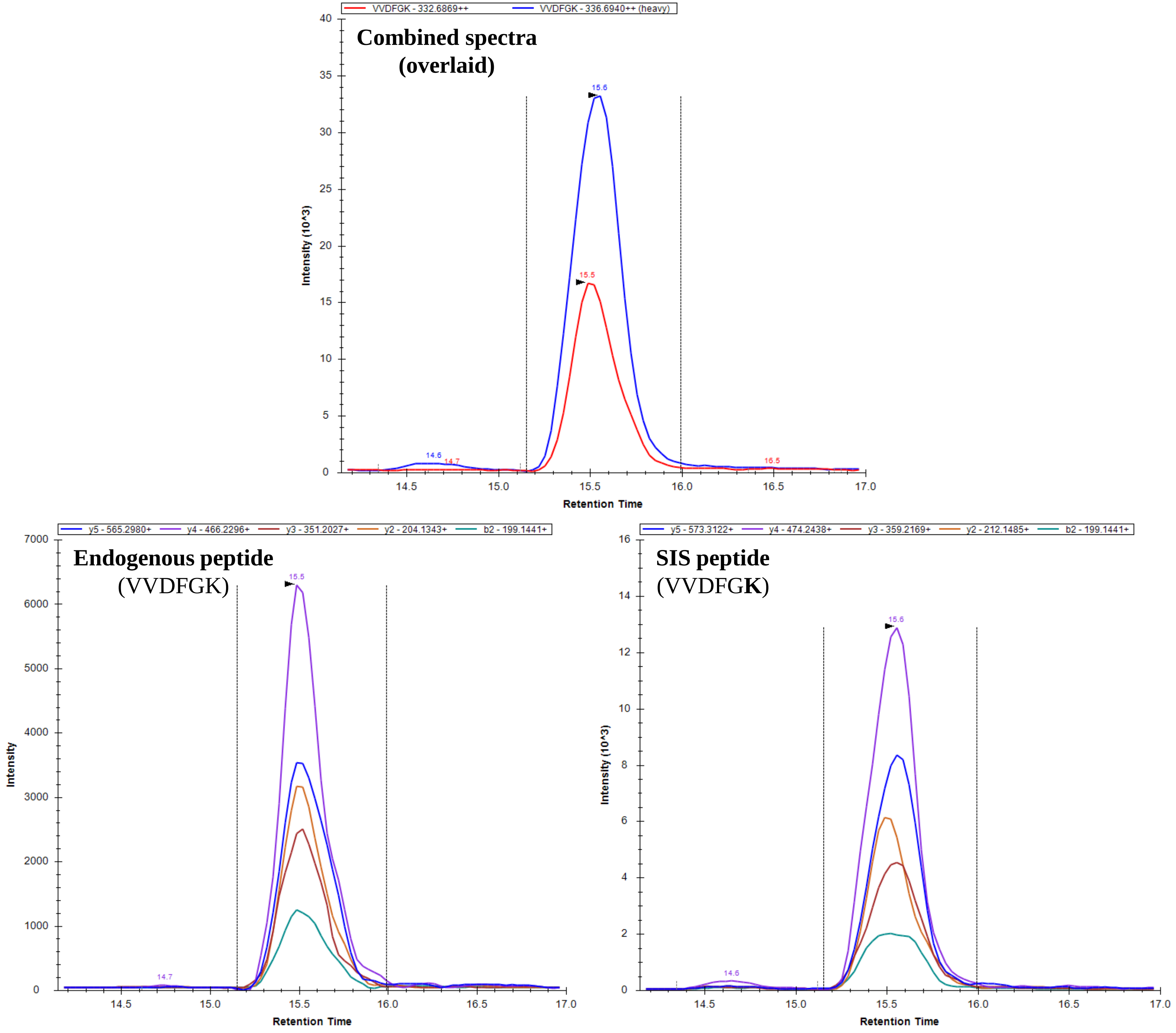

VVDFGK - 332.6869++
VVDFGK - 336.6940++ (heavy)
Combined spectra
(overlaid)
Intensity (10^3)
Retention Time
y5 - 565.2980+
y4 - 466.2296+
y3 - 351.2027+
y2 - 204.1343+
b2 - 199.1441+
Endogenous peptide
(VVDFGK)
Intensity
Retention Time
y5 - 573.3122+
y4 - 474.2438+
y3 - 359.2169+
y2 - 212.1485+
b2 - 199.1441+
SIS peptide
(VVDFGK)
Intensity (10^3)
Retention Time

D)

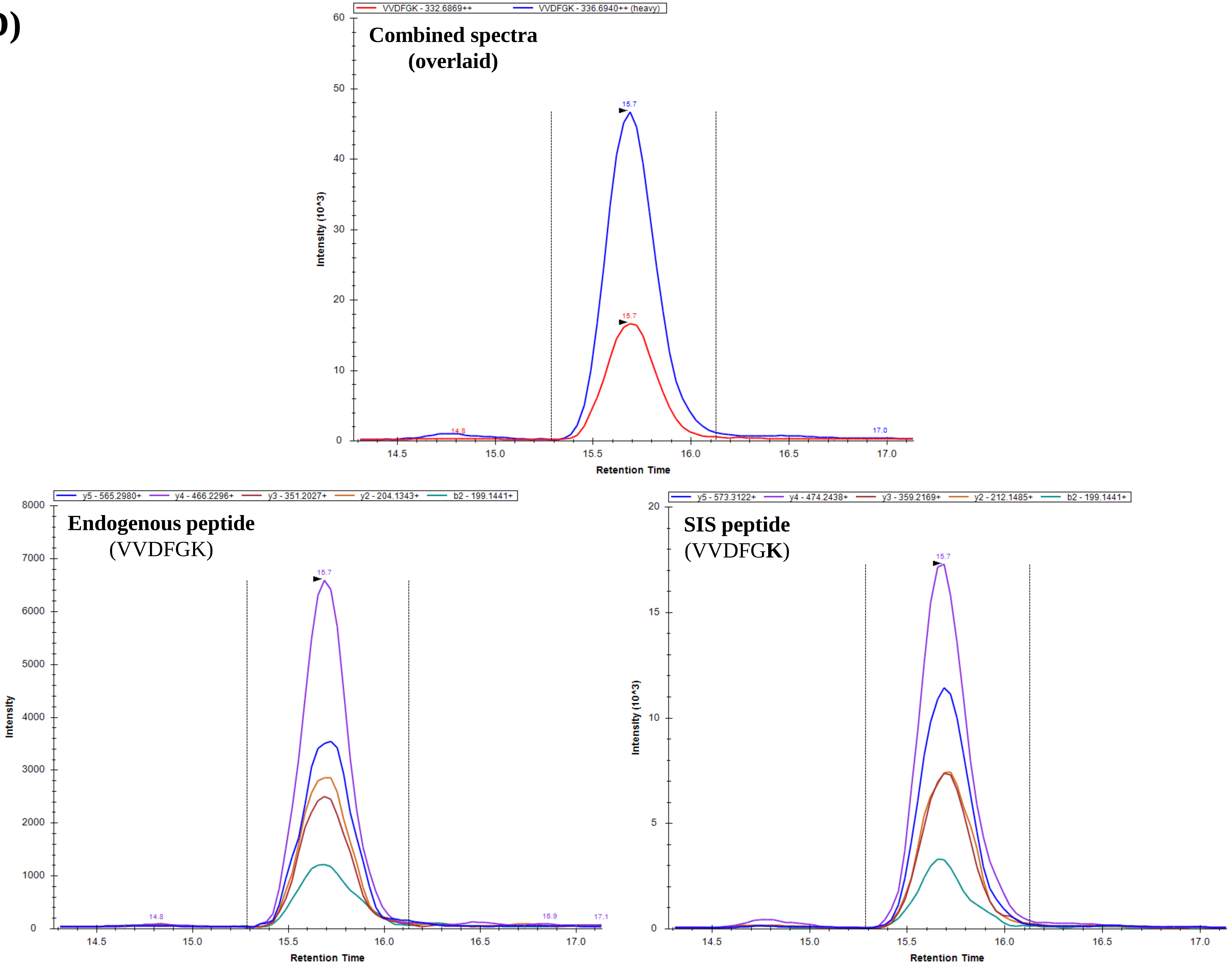

VVDFGK - 332.6869++
VVDFGK - 336.6940++ (heavy)
Combined spectra (overlaid)
Intensity (10^3)
Retention Time
15.7
14.8
17.0
y5 - 565.2980+
y4 - 466.2296+
y3 - 351.2027+
y2 - 204.1343+
b2 - 199.1441+
Endogenous peptide (VVDFGK)
Intensity
Retention Time
16.9
17.1
y5 - 573.3122+
y4 - 474.2438+
y3 - 359.2169+
y2 - 212.1485+
b2 - 199.1441+
SIS peptide (VVDFG**K**)
Intensity (10^3)
Retention Time

E)

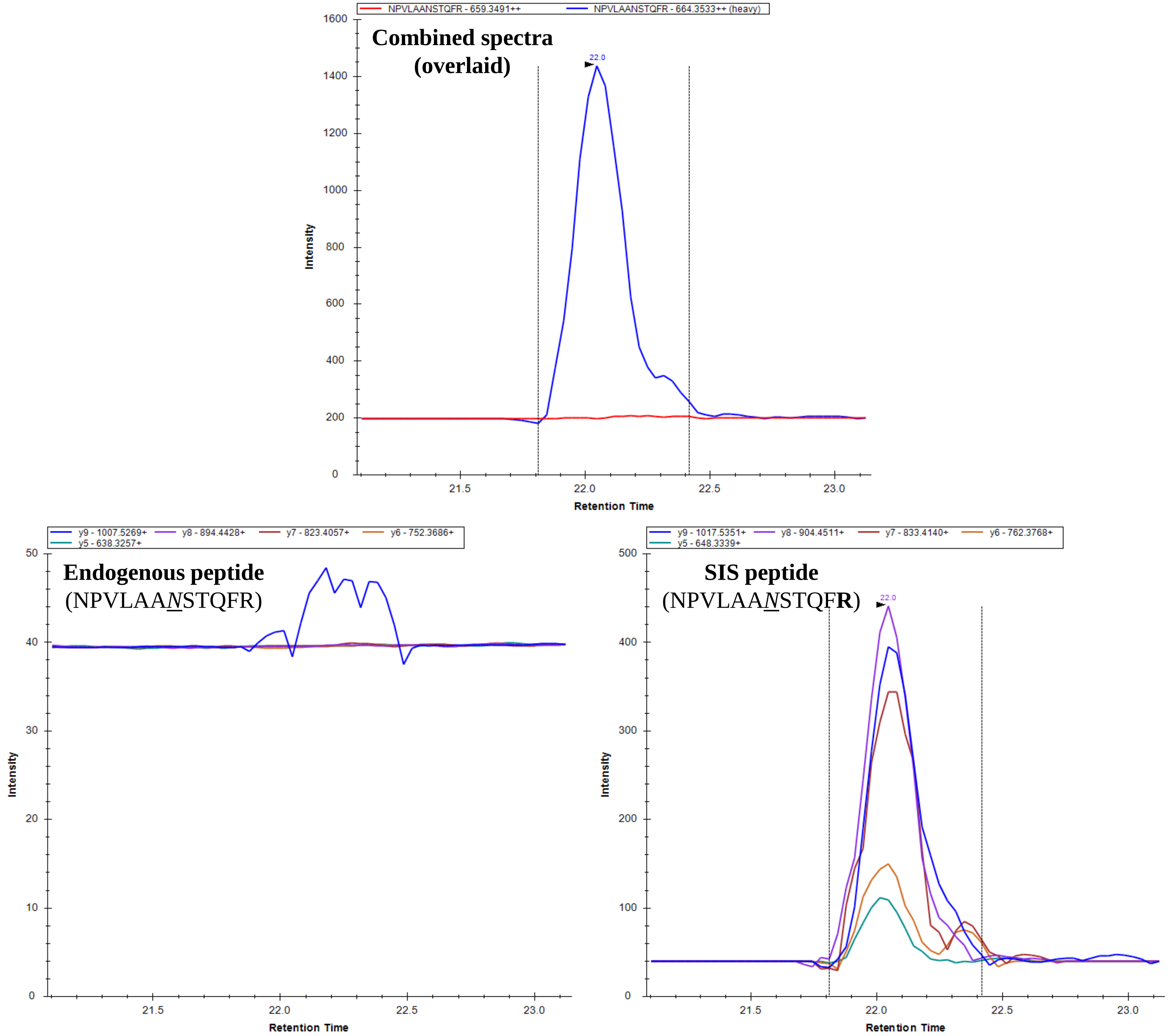

NPVLAANSTQFR - 659.3491++
NPVLAANSTQFR - 664.3533++ (heavy)
Combined spectra
(overlaid)
22.0
Intensity
Retention Time
y9 - 1007.5269+
y8 - 894.4428+
y7 - 823.4057+
y6 - 752.3686+
y5 - 638.3257+
Endogenous peptide
(NPVLAANSTQFR)
Intensity
Retention Time
y9 - 1017.5351+
y8 - 904.4511+
y7 - 833.4140+
y6 - 762.3768+
y5 - 648.3339+
SIS peptide
(NPVLAANSTQFR)
22.0
Intensity
Retention Time

F)

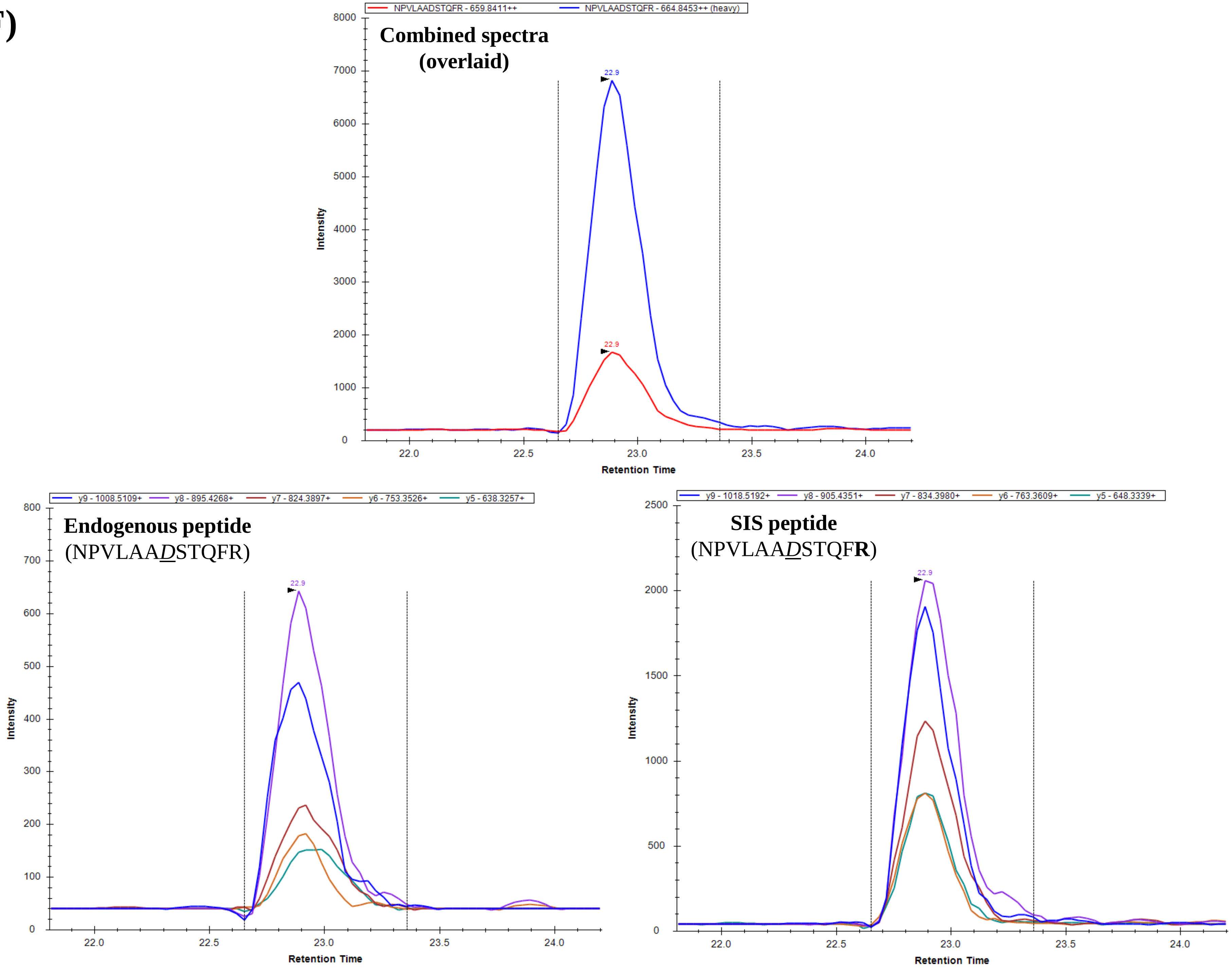

NPVLAADSTQFR - 659.8411++
NPVLAADSTQFR - 664.8453++ (heavy)
Combined spectra (overlaid)
22.9
22.9
Intensity
Retention Time
y9 - 1008.5109+
y8 - 895.4268+
y7 - 824.3897+
y6 - 753.3526+
y5 - 638.3257+
Endogenous peptide (NPVLAADSTQFR)
22.9
Intensity
Retention Time
y9 - 1018.5192+
y8 - 905.4351+
y7 - 834.3980+
y6 - 763.3609+
y5 - 648.3339+
SIS peptide (NPVLAADSTQFR)
22.9
Intensity
Retention Time

G)

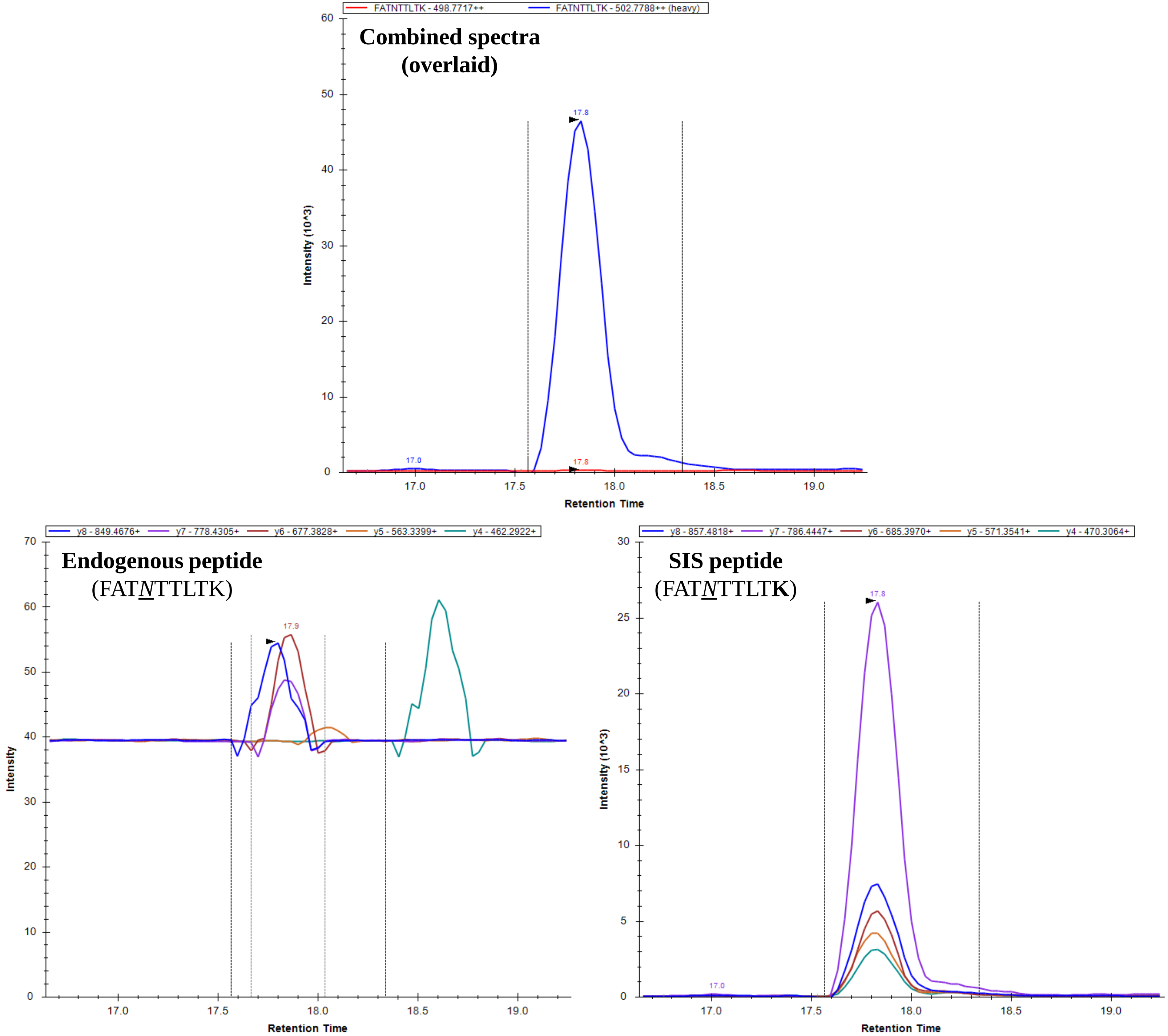
FATNTTLTK - 498.7717++
FATNTTLTK - 502.7788++ (heavy)
Combined spectra
(overlaid)
Intensity (10^3)
Retention Time
17.8
17.0
Endogenous peptide
(FATNTTLTK)
y8 - 849.4676+
y7 - 778.4305+
y6 - 677.3828+
y5 - 563.3399+
y4 - 462.2922+
17.9
Intensity
Retention Time
SIS peptide
(FATNTTLTK)
y8 - 857.4818+
y7 - 786.4447+
y6 - 685.3970+
y5 - 571.3541+
y4 - 470.3064+
17.8
17.0
Intensity (10^3)
Retention Time

H)

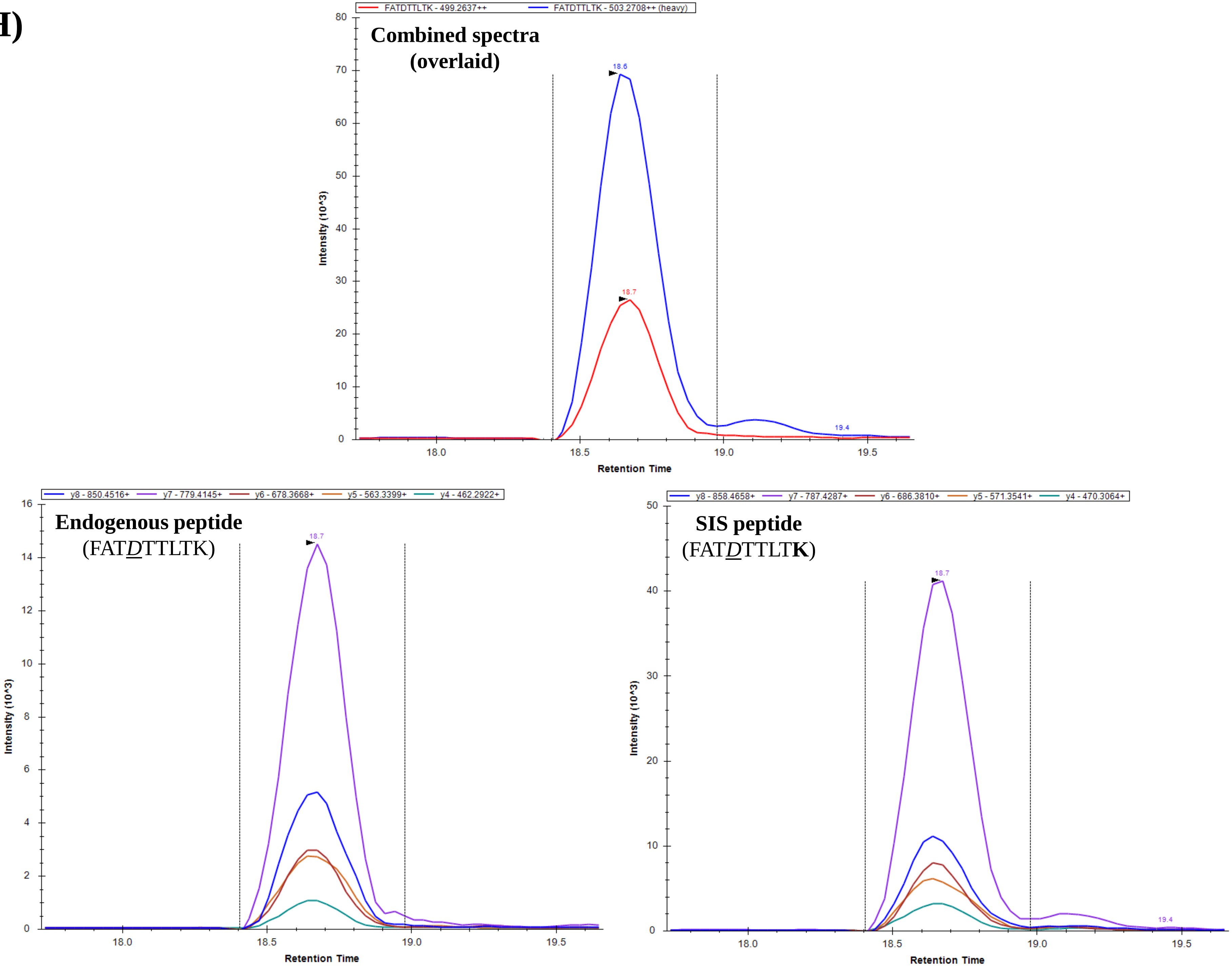

FATDTTLTK - 499.2637++
FATDTTLTK - 503.2708++ (heavy)
Combined spectra
(overlaid)
Intensity (10^3)
Retention Time
18.6
18.7
19.4
y8 - 850.4516+
y7 - 779.4145+
y6 - 678.3668+
y5 - 563.3399+
y4 - 462.2922+
Endogenous peptide
(FAT*D*TTLTK)
y8 - 858.4658+
y7 - 787.4287+
y6 - 686.3810+
y5 - 571.3541+
y4 - 470.3064+
SIS peptide
(FAT*D*TTLT**K**)
