# Supplementary material for: Measurement of Glycosylated Alpha-Fetoprotein Improves Diagnostic Power over the Native Form in Hepatocellular Carcinoma
Source: PLoS One. 2014 Oct 13;9(10):e110366. doi: 10.1371/journal.pone.0110366 (PMC4195728; doi:10.1371/journal.pone.0110366)
Supplement: Figure S4 — (PDF) [file pone.0110366.s004.pdf]

Figure S4. Peak intensities of MRM analysis for target peptides.

Two nonglycopeptides (IEIYSSDDLK, VVDFGK), 2 glycopeptides (NPVLAA*N*STQFR, FAT*N*TTLTK), and 2 deglycopeptides (NPVLAA*D*STQFR, FAT*D*TTLTK) of the standard glycoprotein (INV1) are shown.

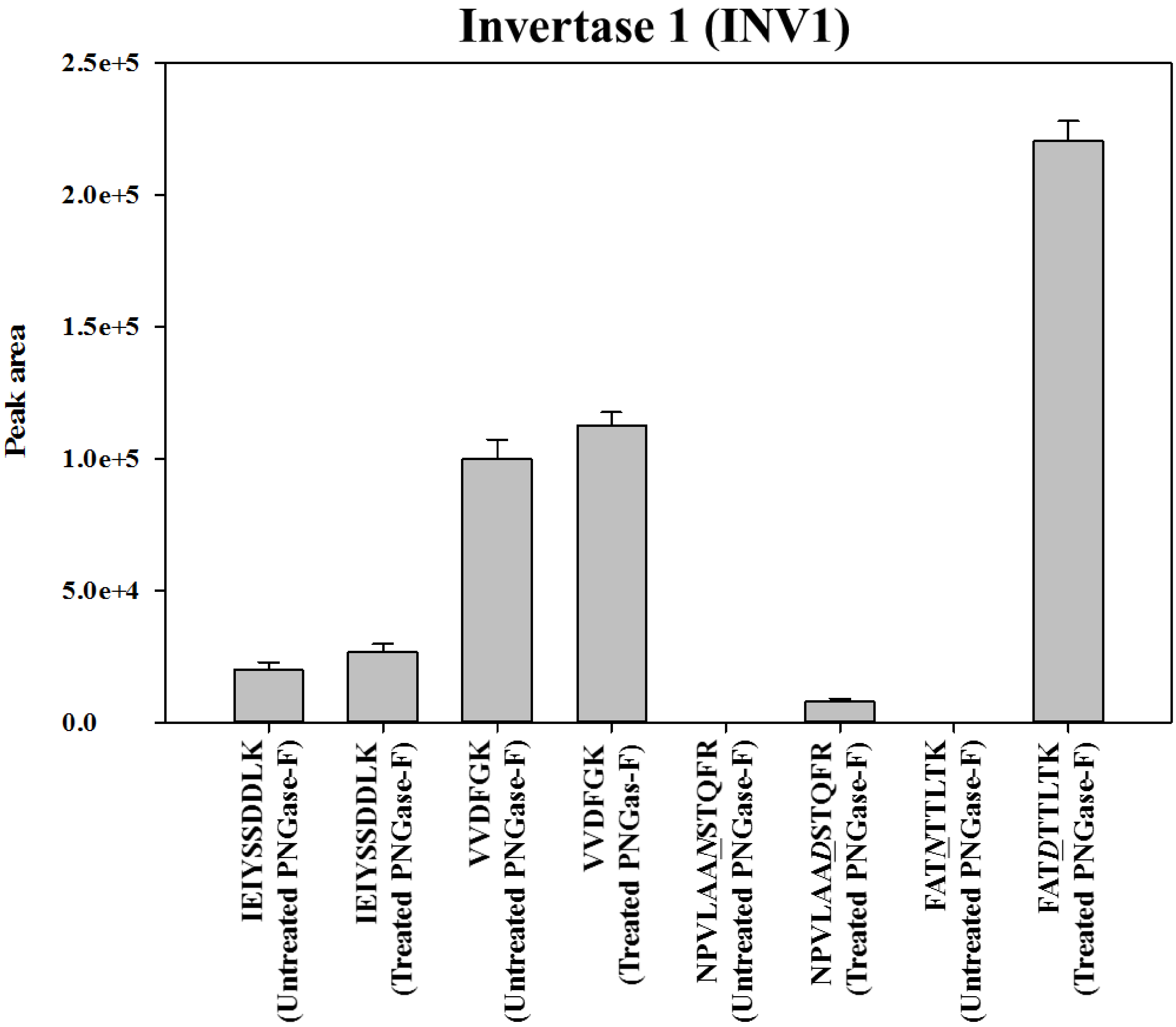
Invertase 1 (INV1)
Peak area
2.5e+5
2.0e+5
1.5e+5
1.0e+5
5.0e+4
0.0
IEIYSSDDLK (Untreated PNGase-F)
IEIYSSDDLK (Treated PNGase-F)
VVDFGK (Untreated PNGase-F)
VVDFGK (Treated PNGas-F)
NPVLAANSTQFR (Untreated PNGase-F)
NPVLAADSTQFR (Treated PNGase-F)
FATNTTLTK (Untreated PNGase-F)
FATDTTLTK (Treated PNGase-F)
