# Supplementary material for: Measurement of Glycosylated Alpha-Fetoprotein Improves Diagnostic Power over the Native Form in Hepatocellular Carcinoma
Source: PLoS One. 2014 Oct 13;9(10):e110366. doi: 10.1371/journal.pone.0110366 (PMC4195728; doi:10.1371/journal.pone.0110366)
Supplement: Figure S5 — (PDF) [file pone.0110366.s005.pdf]

Figure S5. Linear response curves for target peptides.

Two nonglycopeptides (IEIYSSDDLK, VVDFGK), **(A and B)** 2 glycopeptides (NPVLAA*N*STQFR, FAT*N*TTLTK), and 2 deglycopeptides (NPVLAA*D*STQFR, FAT*D*TTLTK) of the standard glycoprotein (INV1) are shown **(C and D)**.

A)

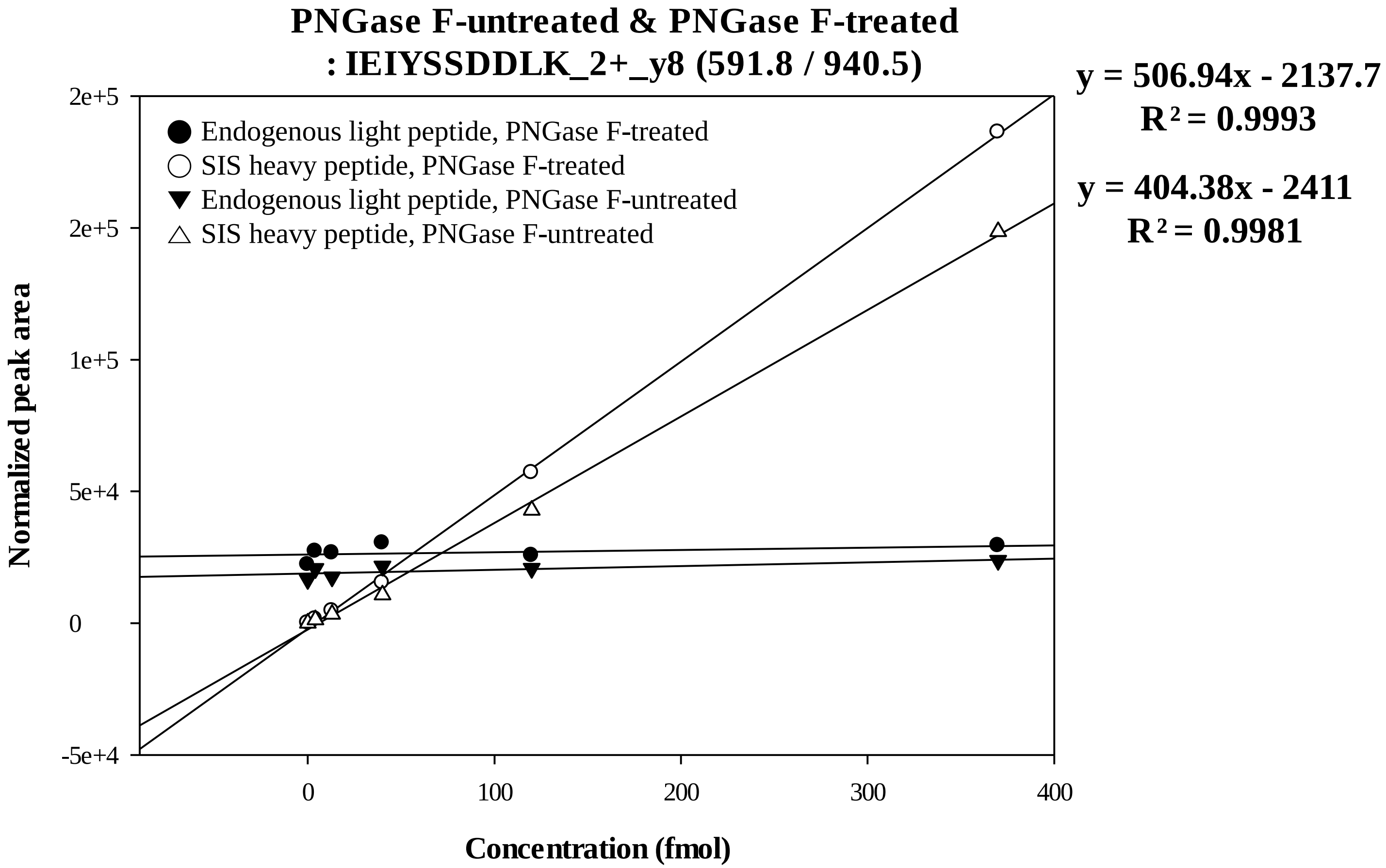

PNGase F-untreated sample

SIS conc. (fmol)	Normalized peak area (fmol) Average	STDEV	CV (%)	Endo conc. (nmol)	Normalized peak area (fmol) Average	STDEV	CV (%)
**0**	0	0	0	**370**	16515	907.5	5.5
**4**	1265.7	405.5	32	**370**	20640.7	1143.2	5.5
**13**	3385.3	302.5	8.9	**370**	17470.3	2239.6	12.8
**40**	10699	2104.3	19.7	**370**	21598	910.4	4.2
**120**	42808.7	3793.7	8.9	**370**	20738	1253.4	6
**370**	148574	2126.4	1.4	**370**	23795.3	838.5	3.5

PNGase F-treated sample

SIS conc. (fmol)	Normalized peak area (fmol) Average	STDEV	CV (%)	Endo conc. (nmol)	Normalized peak area (fmol) Average	STDEV	CV (%)
**0**	0	0	0	**370**	22047.3	342	1.6
**4**	1457	534.5	36.7	**370**	27167.3	1216.9	4.5
**13**	4585	237.8	5.2	**370**	26529.3	3219.1	12.1
**40**	15154	1538.5	10.2	**370**	30355.7	1660.5	5.5
**120**	56990	3247.2	5.7	**370**	25508.3	1855	7.3
**370**	186285	10110.8	5.4	**370**	29249.3	2171.6	7.4

B)

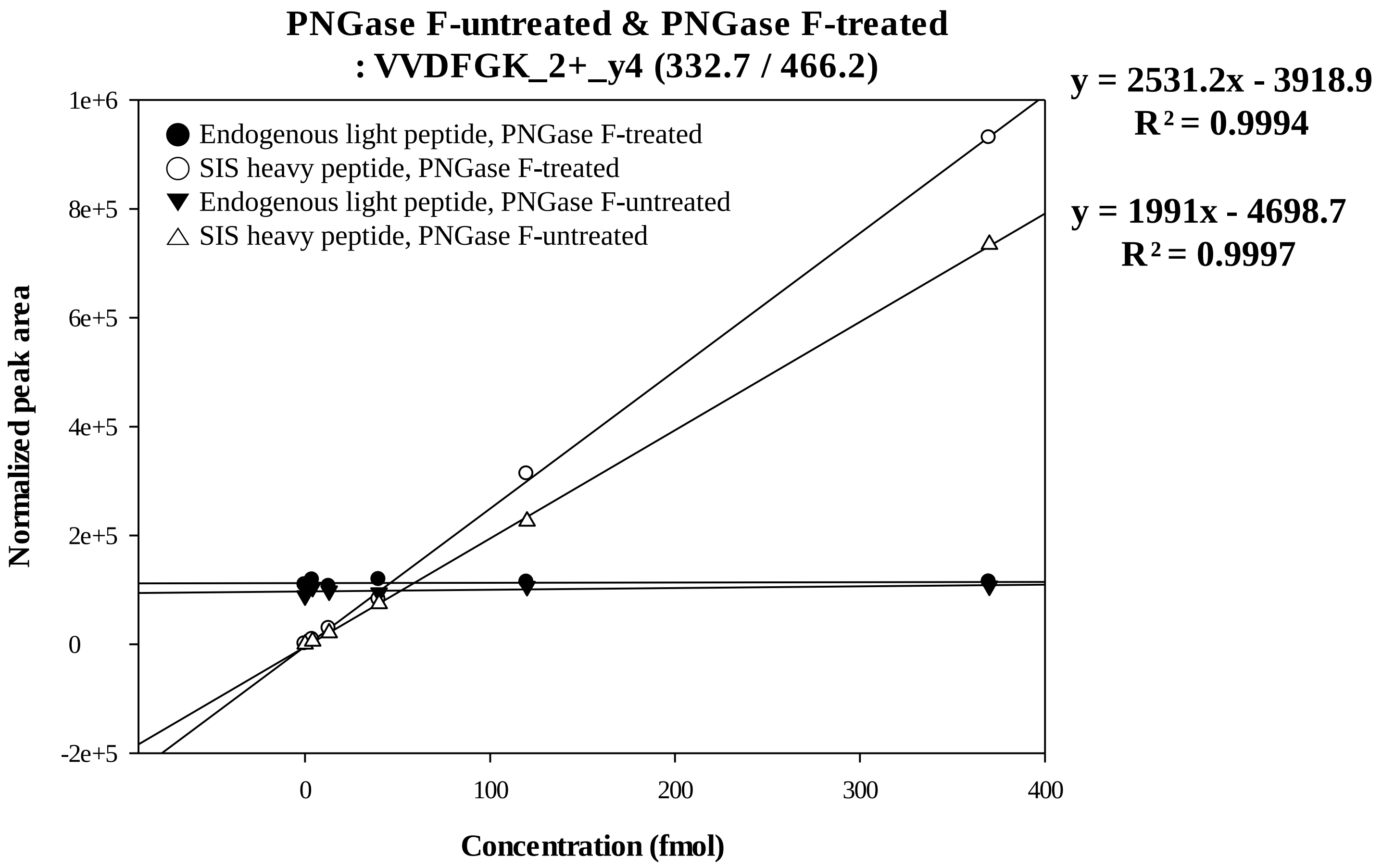

PNGase F-untreated sample

SIS conc. (fmol)	Normalized peak area (fmol)			Endo conc. (nmol)	Normalized peak area (fmol)		
	Average	STDEV	CV (%)		Average	STDEV	CV (%)
**0**	0	0	0	**370**	89205.3	3342.2	3.7
**4**	5565	178.5	3.2	**370**	104520	3551.8	3.4
**13**	20502	2475.7	12.1	**370**	97914.7	3015	3.1
**40**	74142.7	294.1	0.4	**370**	94603.3	6126.7	6.5
**120**	225922.7	13356.7	5.9	**370**	106257.3	3216.8	3
**370**	734734.3	27326.5	3.7	**370**	107177.7	2738.7	2.6

PNGase F-treated sample

SIS conc. (fmol)	Normalized peak area (fmol)			Endo conc. (nmol)	Normalized peak area (fmol)		
	Average	STDEV	CV (%)		Average	STDEV	CV (%)
**0**	0	0	0	**370**	108440	6087.6	5.6
**4**	8115.3	419.8	5.2	**370**	117271.7	3679.3	3.1
**13**	28533.7	1532.7	5.4	**370**	105682	5422.9	5.1
**40**	81770	3646.6	4.5	**370**	118207	4490	3.8
**120**	312418	7315	2.3	**370**	113168.7	466.2	0.4
**370**	930225.7	11860	1.3	**370**	113721	7408	6.5

C)

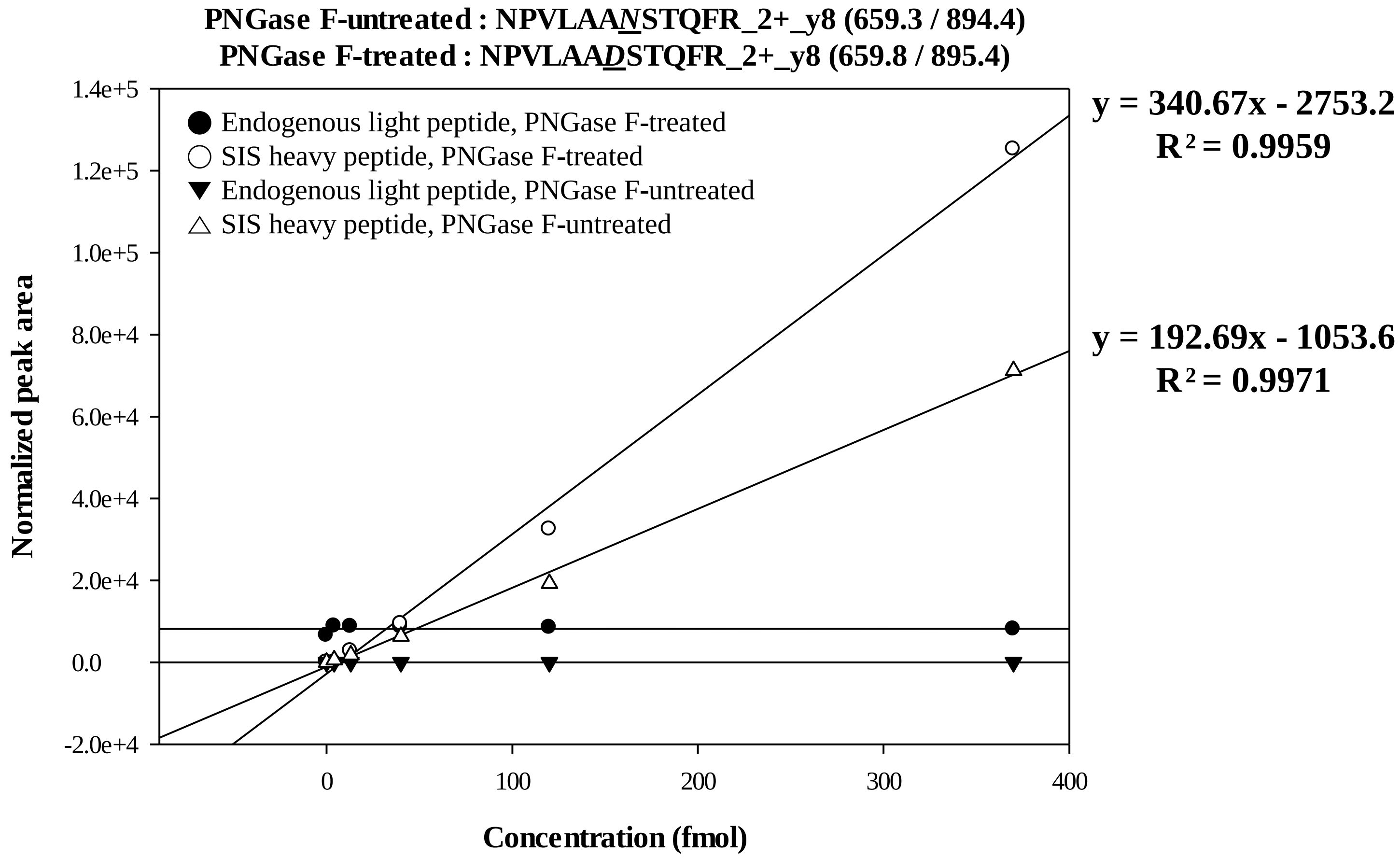

PNGase F-untreated sample

SIS conc. (fmol)	Normalized peak area (fmol)			Endo conc. (nmol)	Normalized peak area (fmol)		
	Average	STDEV	CV (%)		Average	STDEV	CV (%)
**0**	0	0	0	**370**	0	0	0
**4**	567	140.5	24.8	**370**	0	0	0
**13**	1803	439.7	24.4	**370**	0	0	0
**40**	6312.7	985.4	15.6	**370**	0	0	0
**120**	19215	2691.7	14	**370**	0	0	0
**370**	71184.7	2395.1	3.4	**370**	0	0	0

PNGase F-treated sample

SIS conc. (fmol)	Normalized peak area (fmol)			Endo conc. (nmol)	Normalized peak area (fmol)		
	Average	STDEV	CV (%)		Average	STDEV	CV (%)
**0**	0	0	0	**370**	6543	535.2	8.2
**4**	0	0	0	**370**	8770.7	1578.1	18
**13**	2745	992.7	36.2	**370**	8679.7	1077	12.4
**40**	9385	1541.2	16.4	**370**	8590.7	921.5	10.7
**120**	32455	757.8	2.3	**370**	8466.7	546.7	6.5
**370**	125243.3	4397.1	3.5	**370**	8050	1124.4	14

D)

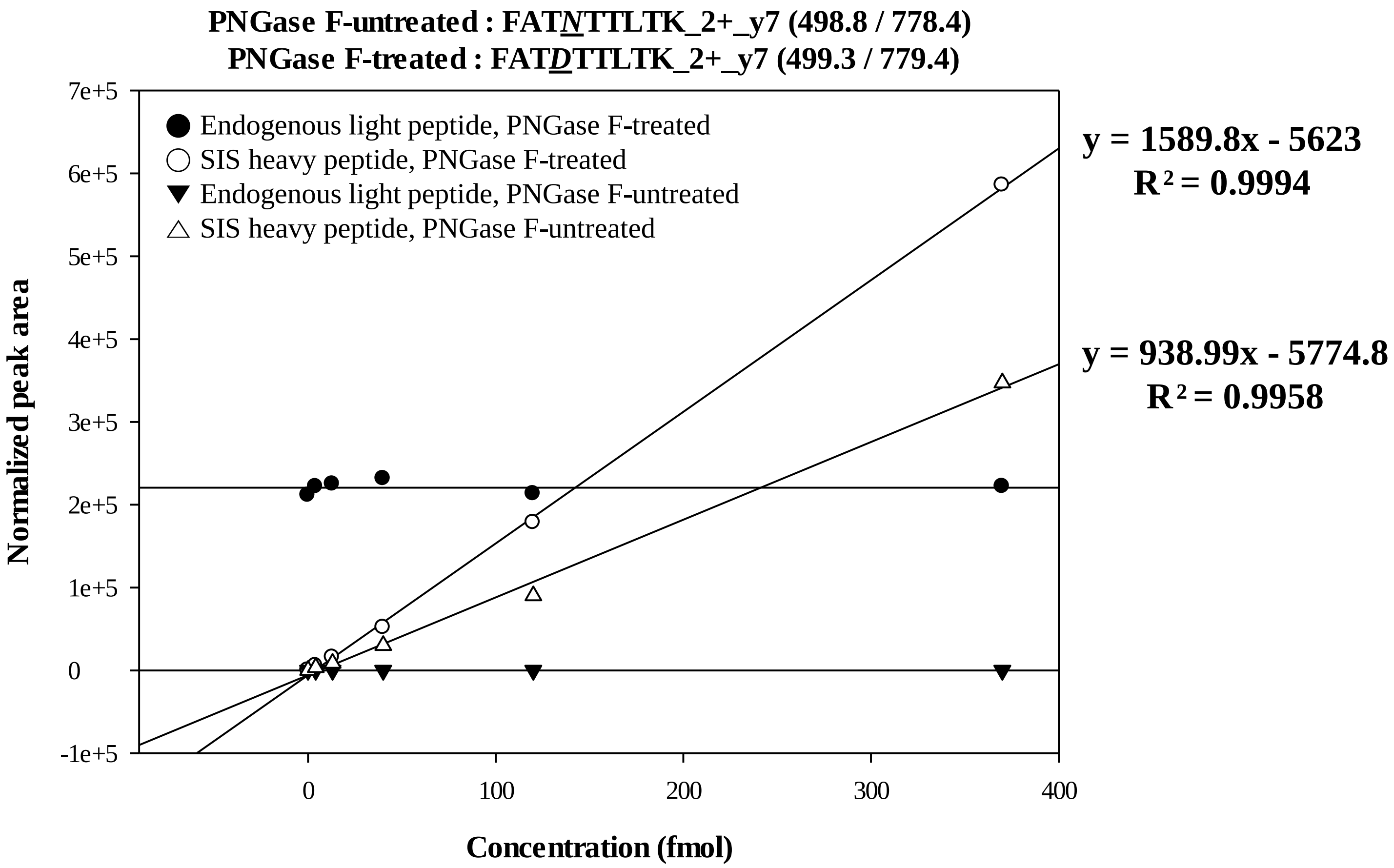

PNGase F-untreated sample

SIS conc. (fmol)	Normalized peak area (fmol)			Endo conc. (nmol)	Normalized peak area (fmol)		
	Average	STDEV	CV (%)		Average	STDEV	CV (%)
**0**	0	0	0	**370**	0	0	0
**4**	3251.7	377.5	11.6	**370**	0	0	0
**13**	8573.7	828.6	9.7	**370**	0	0	0
**40**	29945.7	2731.4	9.1	**370**	0	0	0
**120**	90011	8249.9	9.2	**370**	0	0	0
**370**	347197.7	38220.6	11	**370**	0	0	0

PNGase F-treated sample

SIS conc. (fmol)	Normalized peak area (fmol)			Endo conc. (nmol)	Normalized peak area (fmol)		
	Average	STDEV	CV (%)		Average	STDEV	CV (%)
**0**	0	0	0	**370**	211051	11726.5	5.6
**4**	5316	329.5	6.2	**370**	221477.7	10614.3	4.8
**13**	15605.3	1789.6	11.5	**370**	224643.3	3350.8	1.5
**40**	51313.7	3243.6	6.3	**370**	231082.7	12938	5.6
**120**	178115.7	6992.8	3.9	**370**	212931	19169.8	9
**370**	585547.3	24593.9	4.2	**370**	221705.7	19232.9	8.7
