# Supplementary material for: Measurement of Glycosylated Alpha-Fetoprotein Improves Diagnostic Power over the Native Form in Hepatocellular Carcinoma
Source: PLoS One. 2014 Oct 13;9(10):e110366. doi: 10.1371/journal.pone.0110366 (PMC4195728; doi:10.1371/journal.pone.0110366)
Supplement: Figure S6 — (PDF) [file pone.0110366.s006.pdf]

Figure S6. Confirmation of detectability for nonglycopeptide, glycopeptide, and deglycopeptide.

The nonglycopeptide was treated with PNGase F or not, wherein the endogenous light peptides and corresponding SIS heavy peptides coeluted at the same retention time and the transitions were well overlaid **(A and B)**. The glycopeptide was untreated with PNGase F, and endogenous light peptide that did not coelute with the SIS heavy peptide **(C)**. The deglycopeptide was treated with PNGase F, and the endogenous light peptide coeluted with the SIS heavy peptide **(D)**. The nonglycopeptide and deglycopeptide were detected as coeluted endogenous light and SIS heavy peptides, whereas the glycopeptide was detected only in its SIS heavy peptide form, because the glycopeptide with a glycan could not be identified in this MRM-MS.

A)

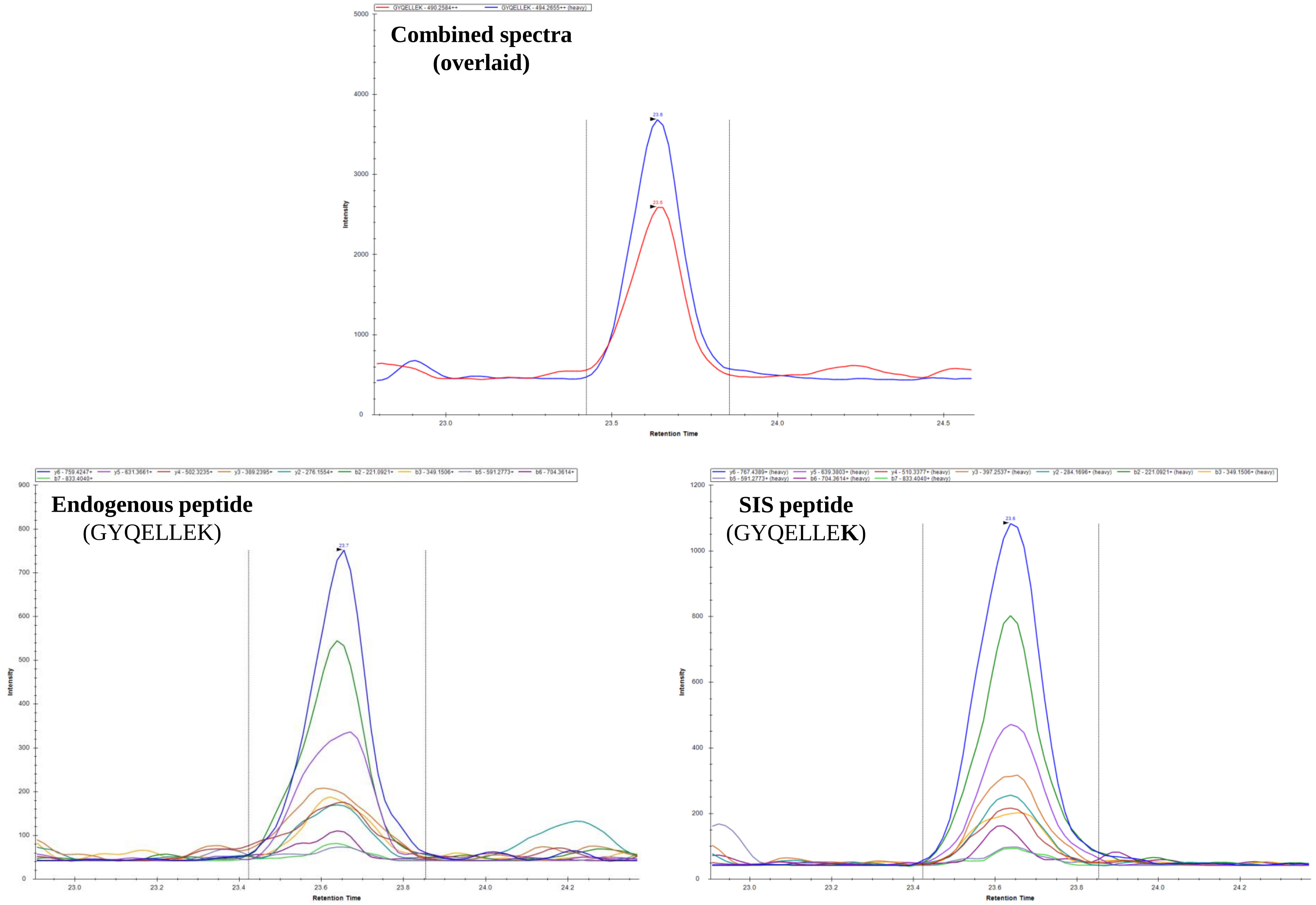
GYQELLEK - 490.2584++
GYQELLEK - 494.2655++ (heavy)
Combined spectra
(overlaid)
Intensity
Retention Time
y6 - 759.4247+
y5 - 631.3661+
y4 - 502.3235+
y3 - 389.2395+
y2 - 276.1554+
b2 - 221.0921+
b3 - 349.1506+
b5 - 591.2773+
b6 - 704.3614+
b7 - 833.4040+
Endogenous peptide
(GYQELLEK)
y6 - 767.4389+ (heavy)
y5 - 639.3803+ (heavy)
y4 - 510.3377+ (heavy)
y3 - 397.2537+ (heavy)
y2 - 284.1696+ (heavy)
b2 - 221.0921+ (heavy)
b3 - 349.1506+ (heavy)
b5 - 591.2773+ (heavy)
b6 - 704.3614+ (heavy)
b7 - 833.4040+ (heavy)
SIS peptide
(GYQELLEK)

B)

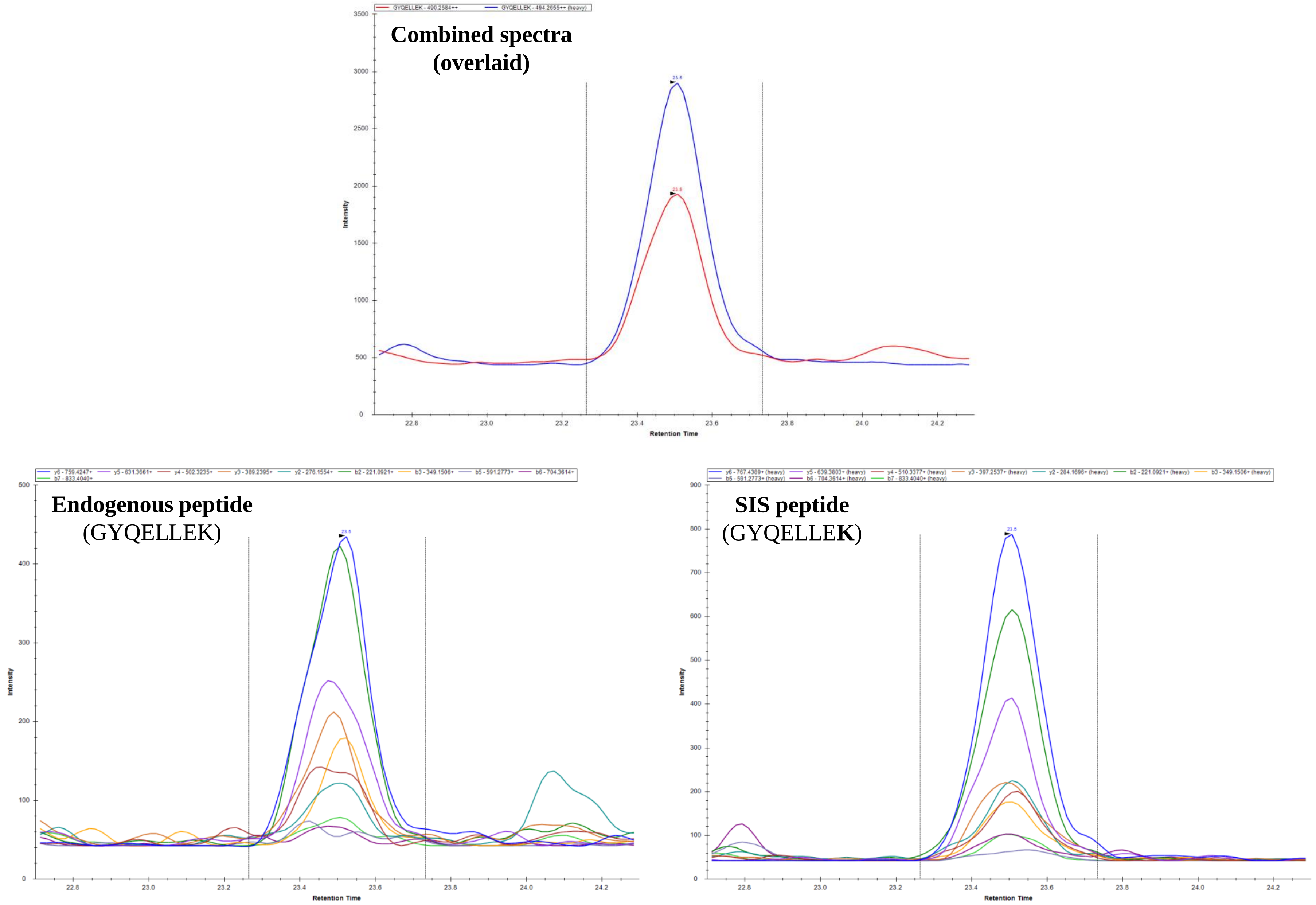
GYQELLEK - 490.2584++
GYQELLEK - 494.2655++ (heavy)
Combined spectra
(overlaid)
Intensity
Retention Time
y6 - 759.4247+
y5 - 631.3661+
y4 - 502.3235+
y3 - 389.2395+
y2 - 276.1554+
b2 - 221.0921+
b3 - 349.1506+
b5 - 591.2773+
b6 - 704.3614+
b7 - 833.4040+
Endogenous peptide
(GYQELLEK)
y6 - 767.4389+ (heavy)
y5 - 639.3803+ (heavy)
y4 - 510.3377+ (heavy)
y3 - 397.2537+ (heavy)
y2 - 284.1696+ (heavy)
b2 - 221.0921+ (heavy)
b3 - 349.1506+ (heavy)
b5 - 591.2773+ (heavy)
b6 - 704.3614+ (heavy)
b7 - 833.4040+ (heavy)
SIS peptide
(GYQELLEK)

C)

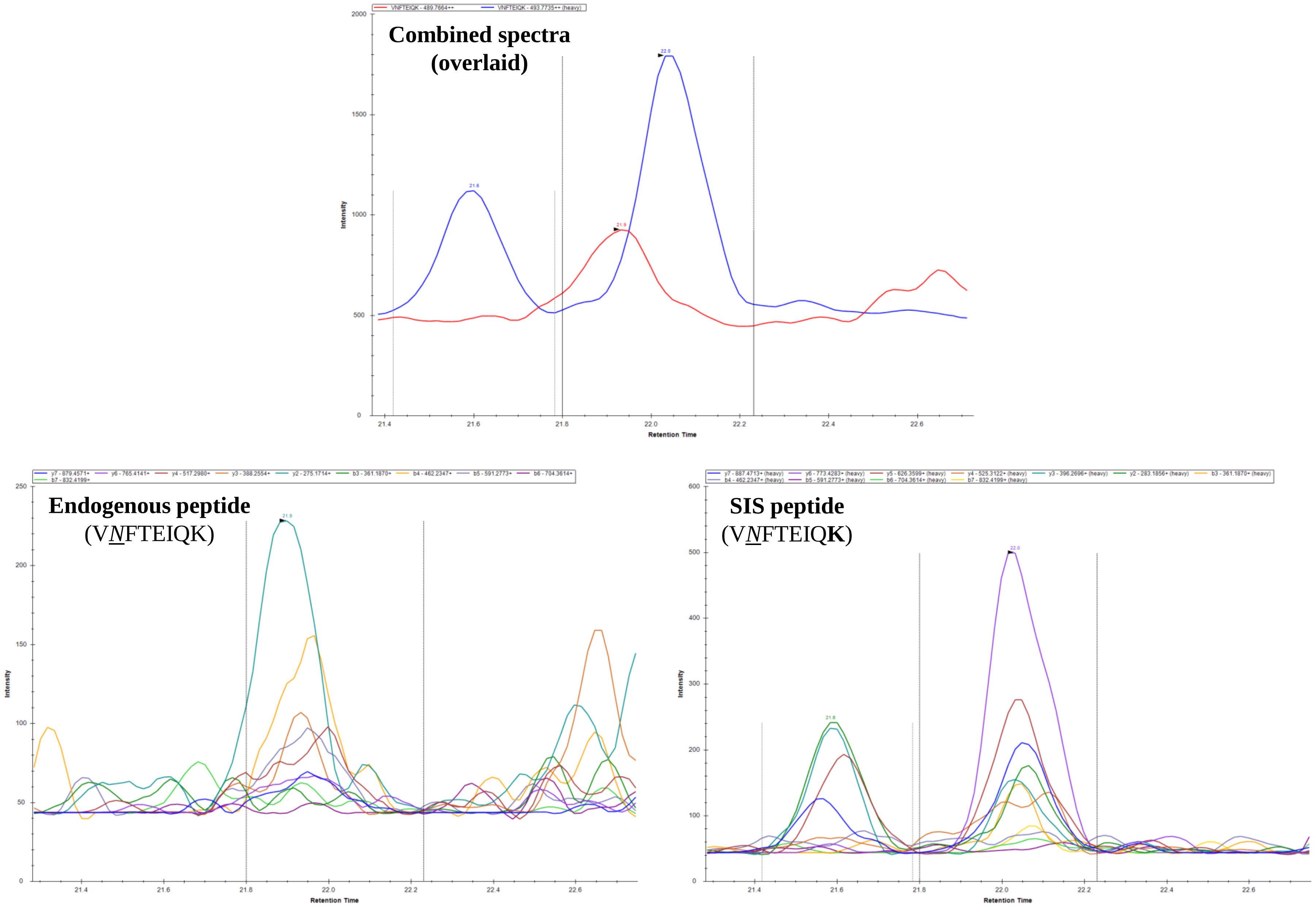
VNFTEIQK - 489.7664++
VNFTEIQK - 493.7735++ (heavy)
Combined spectra
(overlaid)
Intensity
Retention Time
y7 - 879.4571+
y6 - 765.4141+
y4 - 517.2980+
y3 - 388.2554+
y2 - 275.1714+
b3 - 361.1870+
b4 - 462.2347+
b5 - 591.2773+
b6 - 704.3614+
b7 - 832.4199+
Endogenous peptide
(VNFTEIQK)
y7 - 887.4713+ (heavy)
y6 - 773.4283+ (heavy)
y5 - 626.3599+ (heavy)
y4 - 525.3122+ (heavy)
y3 - 396.2696+ (heavy)
y2 - 283.1856+ (heavy)
b3 - 361.1870+ (heavy)
b4 - 462.2347+ (heavy)
b5 - 591.2773+ (heavy)
b6 - 704.3614+ (heavy)
b7 - 832.4199+ (heavy)
SIS peptide
(VNFTEIQK)

D)

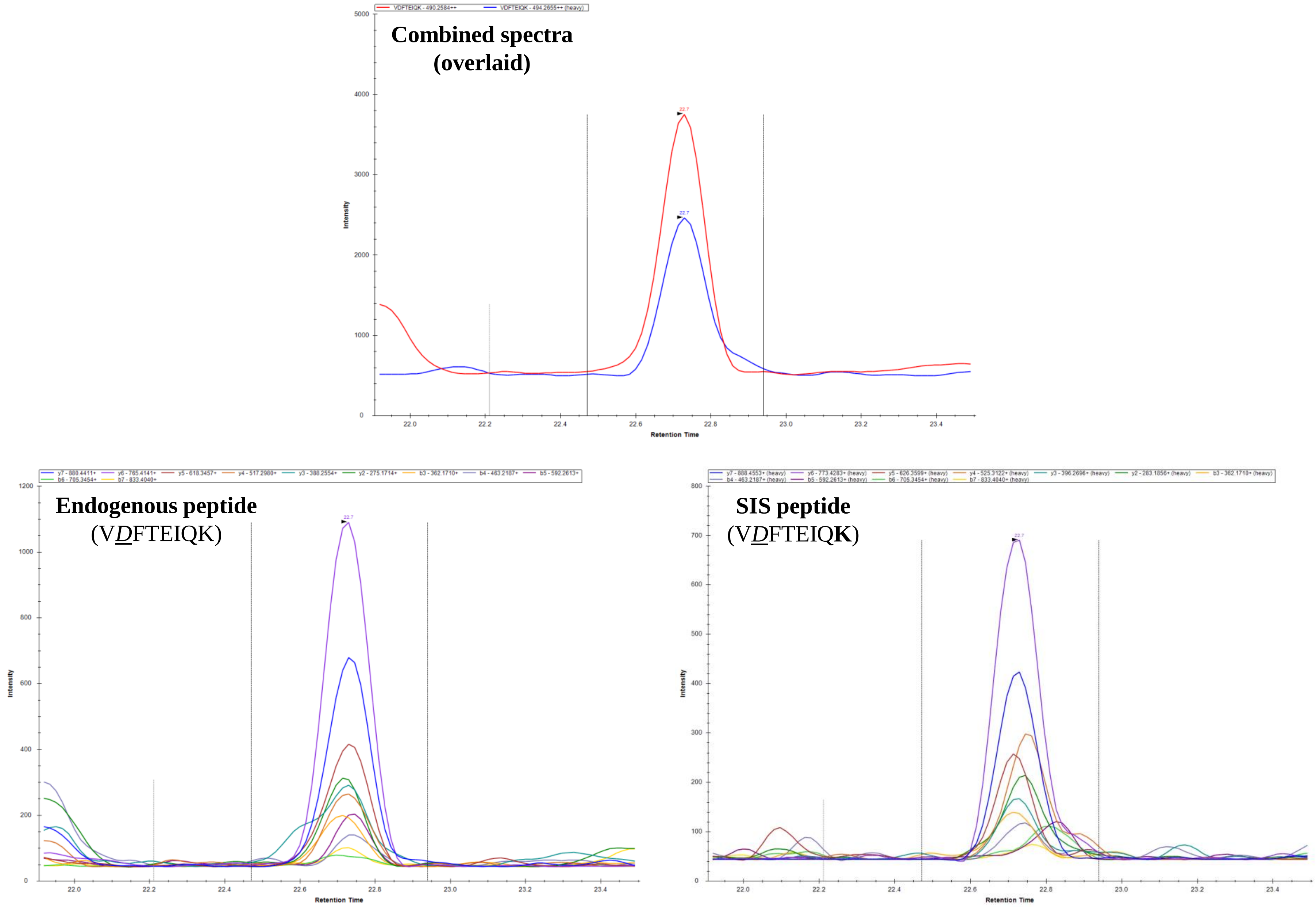
VDFTEIQK - 490.2584++
VDFTEIQK - 494.2655++ (heavy)
Combined spectra (overlaid)
5000
4000
3000
2000
1000
0
Intensity
22.7
22.0
22.2
22.4
22.6
22.8
23.0
23.2
23.4
Retention Time
y7 - 880.4411+
y6 - 765.4141+
y5 - 618.3457+
y4 - 517.2980+
y3 - 388.2554+
y2 - 275.1714+
b3 - 362.1710+
b4 - 463.2187+
b5 - 592.2613+
b6 - 705.3454+
b7 - 833.4040+
Endogenous peptide
(VDFTEIQK)
1200
1000
800
600
400
200
0
Intensity
22.7
22.0
22.2
22.4
22.6
22.8
23.0
23.2
23.4
Retention Time
y7 - 888.4553+ (heavy)
y6 - 773.4283+ (heavy)
y5 - 626.3599+ (heavy)
y4 - 525.3122+ (heavy)
y3 - 396.2696+ (heavy)
y2 - 283.1856+ (heavy)
b3 - 362.1710+ (heavy)
b4 - 463.2187+ (heavy)
b5 - 592.2613+ (heavy)
b6 - 705.3454+ (heavy)
b7 - 833.4040+ (heavy)
SIS peptide
(VDFTEIQK)
800
700
600
500
400
300
200
100
0
Intensity
22.7
22.0
22.2
22.4
22.6
22.8
23.0
23.2
23.4
Retention Time
