# Supplementary material for: Measurement of Glycosylated Alpha-Fetoprotein Improves Diagnostic Power over the Native Form in Hepatocellular Carcinoma
Source: PLoS One. 2014 Oct 13;9(10):e110366. doi: 10.1371/journal.pone.0110366 (PMC4195728; doi:10.1371/journal.pone.0110366)
Supplement: Figure S7 — (PDF) [file pone.0110366.s007.pdf]

Figure S7. Box plots of the nonglycopeptide and deglycopeptide levels measured by MRM in 60 Normal, 35 LC and 60 HCC cases.

Box plots comparing nonglycopeptide **(A)** and deglycopeptide **(B)** in the following patient groups: normal (n = 60), cirrhosis (n = 35), Total HCC (n = 60), stage I (early HCC; n = 30) and stage II and III (late HCC; n = 25). Patients of TNM stage II and III were combined because of the low number of samples. The ends of the boxes define the 25th and 75th percentiles. A line inside the box represents the mean, and error bars define the 10th and 90th percentiles. Points beyond the 10th and 90th percentiles are also displayed. For definitions of the HCC subgroups, see text. Both the normal and cirrhosis groups are significantly different from the total HCC group and HCC subgroup (stage I and stage II, III) at *P*-value ≤ 0.05. The serum values of nonglycopeptide and deglycopeptide were significantly increased in HCC compared with those in normal and cirrhosis group (*P*-value ≤ 0.05). However, there was no significant difference between normal subjects versus cirrhosis and stage I HCC versus stage II and III HCC (*P*-value > 0.05).

A)

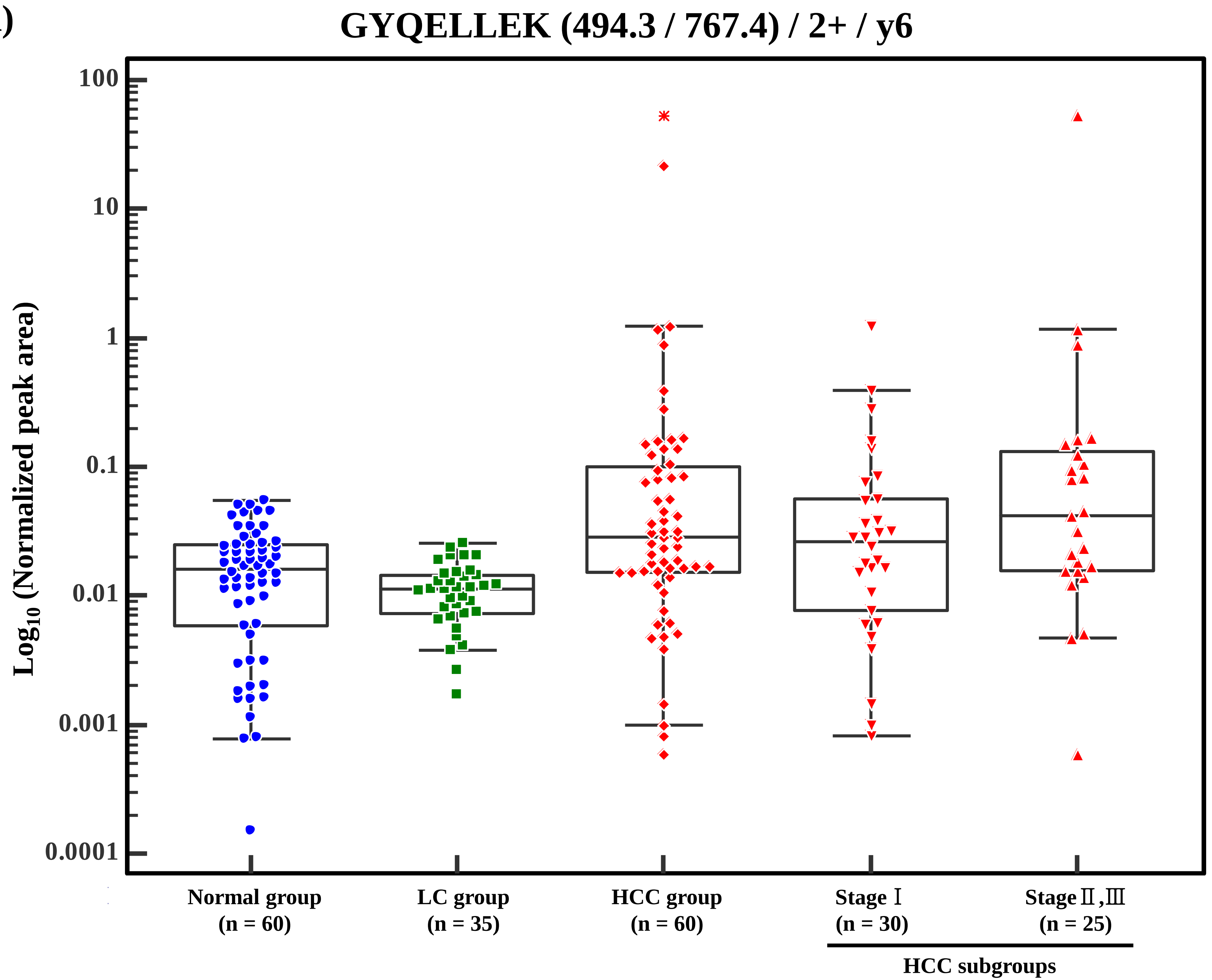
GYQELLEK (494.3 / 767.4) / 2+ / y6
Log10 (Normalized peak area)
100
10
1
0.1
0.01
0.001
0.0001
Normal group
(n = 60)
LC group
(n = 35)
HCC group
(n = 60)
Stage Ⅰ
(n = 30)
Stage Ⅱ,Ⅲ
(n = 25)
HCC subgroups

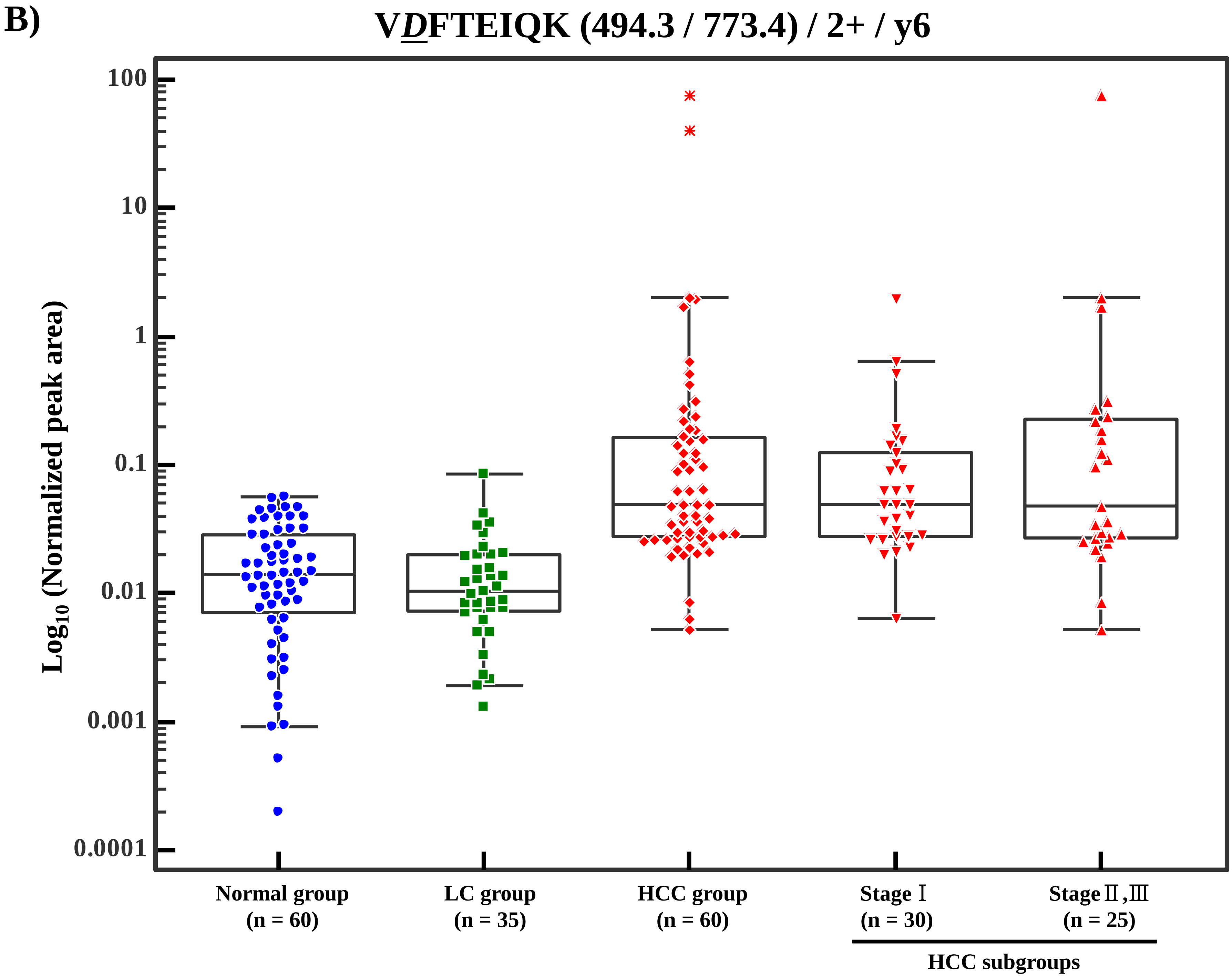
B)
VDFTEIQK (494.3 / 773.4) / 2+ / y6
Log10 (Normalized peak area)
100
10
1
0.1
0.01
0.001
0.0001
Normal group
(n = 60)
LC group
(n = 35)
HCC group
(n = 60)
Stage Ⅰ
(n = 30)
Stage Ⅱ,Ⅲ
(n = 25)
HCC subgroups
